# Socioeconomic Inequalities in the External Exposome in European Cohorts: The EXPANSE Project

**DOI:** 10.1021/acs.est.4c01509

**Published:** 2024-09-05

**Authors:** Apolline Saucy, Fabián Coloma, Sergio Olmos, Christofer Åström, Natalia Blay, Jolanda M.A. Boer, Payam Dadvand, Jeroen de Bont, Rafael de Cid, Kees de Hoogh, Konstantina Dimakopoulou, Ulrike Gehring, Anke Huss, Dorina Ibi, Klea Katsouyanni, Gerard Koppelman, Petter Ljungman, Erik Melén, Mark Nieuwenhuijsen, Federica Nobile, Annette Peters, Regina Pickford, Roel Vermeulen, Danielle Vienneau, Jelle Vlaanderen, Kathrin Wolf, Zhebin Yu, Evangelia Samoli, Massimo Stafoggia, Cathryn Tonne, on behalf of the EXPANSE Project Team

**Affiliations:** † ISGlobal, 08003 Barcelona, Spain; ‡ Universitat Pompeu Fabra (UPF), 08003 Barcelona, Spain; § CIBER Epidemiología y Salud Pública (CIBERESP), 28029 Madrid, Spain; ∥ Department of Public Health and Clinical Medicine, Umeå University, 901 87 Umeå, Sweden; ⊥ Genomes for Life-GCAT Lab, German Trias i Pujol Research Institute (IGTP), 08916 Badalona, Spain; # National Institute for Public Health and the Environment, 3721 Bilthoven, The Netherlands; ∇ Institute of Environmental Medicine, Karolinska Institutet, 171 77 Stockholm, Sweden; ○ Swiss Tropical and Public Health Institute Basel, 4123 Allschwil, Switzerland; ◆ University of Basel, 4001 Basel, Switzerland; ¶ Department of Hygiene, Epidemiology and Medical Statistics, Medical School, National and Kapodistrian University of Athens, 115 27 Athens, Greece; & Institute for Risk Assessment Sciences (IRAS), Utrecht University, 3584 Utrecht, The Netherlands; ● MRC Centre for Environment and Health, School of Public Health, Imperial College London, London W2 1PG, U.K.; ◊ Department of Pediatric Pulmonology, Beatrix Children’s Hospital, University Medical Center Groningen, University of Groningen, 9713 Groningen, The Netherlands; ▲ Groningen Research Institute for Asthma and COPD, University of Groningen, 9713 Groningen, The Netherlands; □ Department of Cardiology, Danderyd Hospital, 171 77 Stockholm, Sweden; ^ Department of Clinical Sciences and Education, Södersjukhuset, Karolinska Institutet, 171 77 Stockholm, Sweden; ¢ Sachś Children and Youth Hospital, Södersjukhuset, 118 61 Stockholm, Sweden; + Department of Epidemiology, Lazio Region Health Service/ASL Roma 100147 Rome, Italy; $ Institute of Epidemiology, Helmholtz Zentrum München, German Research Center for Environmental Health, 85764 Neuherberg, Germany; ∠ IBE, Faculty of Medicine, Ludwig-Maximilians-Universität, 81377 Munich, Germany

**Keywords:** external exposome, socioeconomic determinants, European cohorts, environmental health equity

## Abstract

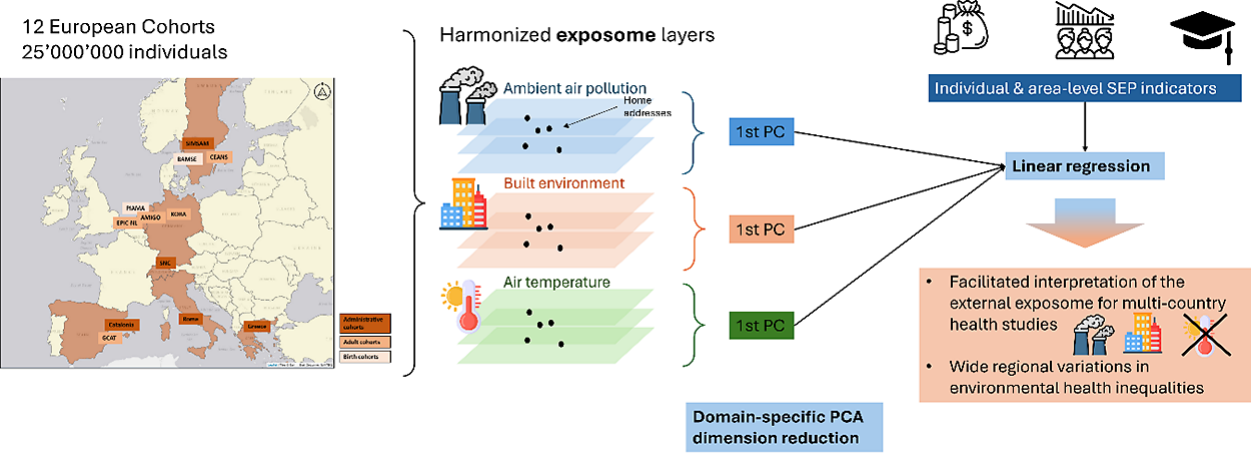

Socioeconomic inequalities in the exposome have been found to be complex and highly context-specific, but studies have not been conducted in large population-wide cohorts from multiple countries. This study aims to examine the external exposome, encompassing individual and environmental factors influencing health over the life course, and to perform dimension reduction to derive interpretable characterization of the external exposome for multicountry epidemiological studies. Analyzing data from over 25 million individuals across seven European countries including 12 administrative and traditional cohorts, we utilized domain-specific principal component analysis (PCA) to define the external exposome, focusing on air pollution, the built environment, and air temperature. We conducted linear regression to estimate the association between individual- and area-level socioeconomic position and each domain of the external exposome. Consistent exposure patterns were observed within countries, indicating the representativeness of traditional cohorts for air pollution and the built environment. However, cohorts with limited geographical coverage and Southern European countries displayed lower temperature variability, especially in the cold season, compared to Northern European countries and cohorts including a wide range of urban and rural areas. The individual- and area-level socioeconomic determinants (i.e., education, income, and unemployment rate) of the urban exposome exhibited significant variability across the European region, with area-level indicators showing stronger associations than individual variables. While the PCA approach facilitated common interpretations of the external exposome for air pollution and the built environment, it was less effective for air temperature. The diverse socioeconomic determinants suggest regional variations in environmental health inequities, emphasizing the need for targeted interventions across European countries.

## Abstract 2

There is limited evidence on geographical disparities in the exposome and the role of the social environment. This study shows regional differences in Europe’s external exposome, with Southern regions facing higher ambient air pollution. Socioeconomic disparities vary geographically, with implications for multicountry environmental health studies.

## Introduction

While the social environment is a powerful influencer of environmental exposures, it is still poorly considered in the exposome literature compared to other exposome domains such as the physicochemical and built environment.^1^ Many conceptual (e.g., biological mechanisms) and practical (e.g., data availability and comparability of measures across data sets) issues related to incorporating social and socioeconomic variables in exposome studies remain underdeveloped.^1^ Studies have also varied in whether they consider socioeconomic variables such as socioeconomic position (SEP) as coexposures, confounders, or effect modifiers of other exposome domains in relation to health.^1−4^

Previous studies characterizing socioeconomic inequalities in the exposome have reported heterogeneous SEP–exposome relationships, indicating that these relationships are complex and highly context-specific. Sum et al. reported that only 14 out of 134 maternal exposures as part of the external and internal pregnancy exposome were associated with at least one SEP indicator in a mother–child cohort in Singapore, a setting with minimal geographic patterns in SEP.^5^ Moccia and colleagues reported that children living in the area of Turin, Italy, with lower SEP were exposed to lower air pollution concentrations and higher amounts of green space but were more exposed to unhealthy lifestyles and diet.^6^ In a multicohort, multicountry study, Robinson et al. observed that the relationship between SEP and hazardous urban exposome exposures during pregnancy varied considerably across nine urban areas in Europe.^3^ Heterogeneity in SEP–exposome relationships presents challenges in the harmonized analysis and interpretation of SEP as a confounder or effect modifier in multicountry studies of the influence of the exposome on health. Available evidence comes largely from traditional cohorts, which typically have rich individual-level data to allow for better control for confounding but are often selected toward higher socioeconomic groups of the target population and, thus, have potentially limited representativeness. Further evidence is needed from large population-based data sets on how exposome distributions and SEP–exposome relationships differ across geographic contexts and in traditional versus population-wide cohorts.

Our aim was to characterize the distribution of the external exposome in multiple European cohorts included in the EXPANSE project (EXposome Powered tools for healthy living in urbAN SEttings) according to the geographical extent, cohort type, and individual- and area-level SEP and to conduct dimension reduction techniques to derive interpretable definitions of the external exposome applicable for multicountry epidemiological studies. We took an agnostic approach to characterizing socioeconomic inequalities in the external exposome to assess whether the magnitude and direction of inequalities differed geographically and across cohort types and to inform how best to consider SEP (e.g., coexposure, confounder, and modifier) in subsequent studies of the external exposome and health in these cohorts.

## Materials and Methods

### Study Population

**Figure 1:**
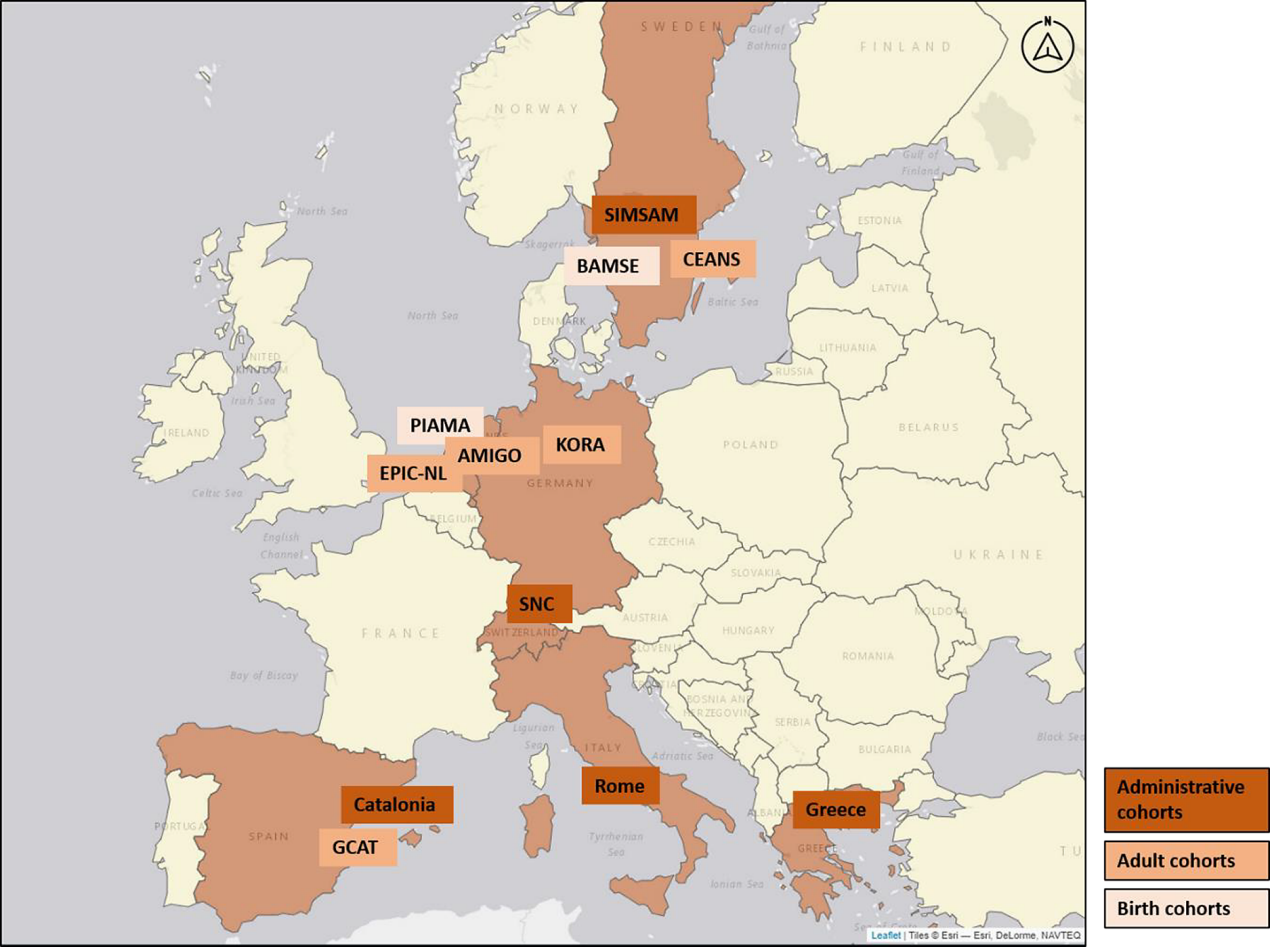
Geographical distribution of the included cohorts.

We included data from 12 cohorts from seven European countries participating in the EXPANSE project (Figure 1). Cohorts included five large administrative cohorts (Switzerland, Rome, Sweden, Greece, and Catalonia) and seven traditional “recruited” cohorts (five adult cohorts: EPIC-NL, AMIGO, GCAT, KORA, and CEANS and two birth cohorts: PIAMA and BAMSE). The administrative cohorts cover the population at the national (e.g., Swiss national cohort (SNC), Swedish national cohort (SIMSAM), and Greek national cohort (Greece)) or regional/city level (e.g., Catalonia and Rome). They also overlap with several of the traditional cohorts, allowing for the evaluation of differences between recruited samples (traditional cohorts) and the target general population. All home addresses at the baseline were geolocated and linked to external exposome data developed in the EXPANSE project. We collected age at the baseline as well as cohort-specific individual- and area-level socioeconomic indicators (Table 1). For the adults and administrative cohorts, we included all individuals 18 years or older with available address and exposure data at the baseline. The minimum age was 37 years in the Swedish and Greek national cohorts and 30 years in Rome. For the birth cohorts, we used maternal addresses and baseline data at the time of birth.

**Table 1:** Summary of Included Cohorts Including Sample Size, Geographical Coverage, Age, and SEP Indicators

cohort type	**cohort**^a^	**country and geographic coverage**	***N*.**	**mean age (baseline)**	**individual SEP indicator**	**area-level SEP indicator**
administrative	Switzerland (SNC)	Switzerland, national	6,162,375	47.7	Swiss index	Swiss index, mean at the community level (*n* = 2583)
	Rome	Italy, metropolitan area	1,737,570	56.4	education	reverse deprivation index at the census block level
	Greece	Greece, national	6,121,421	60.3		% tertiary education at the municipality level or square block level for cities with a population >100,000
	Sweden (SIMSAM)	Sweden, national	4,886,633	58.9	education	mean income at the district level
	Catalonia	Spain, autonomous region	4,954,486	49.4	income	socioeconomic index (PSCA) at the primary care service area level
adult	EPIC-NL	The Netherlands, metropolitan area	33,182	53.3	education	inverse % of low income at the neighborhood level
	AMIGO	The Netherlands, country-wide	13,709	50.7	education	inverse % of low income at the neighborhood level (“Buurt”)
	GCAT	Spain, Catalan autonomous region	17,423	51.0	family income	inverse deprivation index at the census district level
	KORA	Germany, metropolitan area	8725	49.7	education	mean income
	CEANS	Sweden, metropolitan area	20,108	56.4	education	mean income at the small area market statistics level (SAMS)
birth	BAMSE	Sweden	3986	0	household SEP	median income at SAMS
	PIAMA	The Netherlands	3657	0	parental education	SEP score at the neighborhood level (“Buurt”)

a Abbreviations: SNC, Swiss national cohort; SIMSAM, Swedish Initiative for Research on Microdata in the Social and Medical Sciences; EPIC-NL, European Prospective Investigation into Cancer and Nutrition—the Netherlands; AMIGO, Occupational and Environmental Health Cohort Study; GCAT, GCAT|Genomes for Life Cohort study of the Genomes of Catalonia; KORA, Cooperative Health Research in the Augsburg Region; CEANS, Cardiovascular Effects of Air pollution and Noise in Stockholm study; BAMSE, Children, Allergy, Milieu, Stockholm, Epidemiology; PIAMA, Prevention and Incidence of Asthma and Mite Allergy.

### External Exposome Data

The external exposome was characterized using a range of exposure surfaces available for the whole European region using a harmonized exposure assessment protocol.^7^ For this analysis, we considered three a priori defined “domains” of the external exposome: air pollution, the built environment, and air temperature. Ambient air pollution surfaces (particulate matter with a diameter less than 2.5 μm: PM2.5; nitrogen dioxide: NO_2_; black carbon: BC; and warm season ozone: O_3_) were developed as part of the ELAPSE project using a hybrid land use regression approach at 100 × 100 m resolution. The data used for the models include air pollution monitoring data (for model building and validation), satellite observations, dispersion model estimates, land use, and traffic data.^8^ Built environment variables (NDVI (normalized difference vegetation index), impervious surfaces, and distance to blue spaces) were calculated specifically for the EXPANSE project. The NDVI was derived from the vegetation indices (MOD13Q1) product of the Terra Moderate Resolution Imaging Spectroradiometer (MODIS) with 250 m × 250 m resolution.^9^ The straight-line distance to the nearest blue space was assessed using the EU-Hydro map developed by the CLMS.^10^ Gray (i.e., built-up) spaces were estimated using imperviousness density (IMD) maps.^11^ Daily temperature data were available at 11 × 11 km resolution from the European Centre for Medium-Range Weather Forecasts (ECMWF) ERA5-Land reanalysis data set for 2010.^12^ For this analysis, we derived the average temperature and standard deviation for cold (October to March) and warm seasons (April to September). Data sources and exposure periods are displayed in Supplementary Table S1. Exposome values were extracted for all individuals at the baseline home location.

### Socioeconomic Indicators

We collected cohort-specific indicators for individual- and area-level SEP. Education and income were the most common SEP indicators available at the individual level across cohorts. At the area level, the most commonly available indicators were income, deprivation indices, and other composite SEP indicators available at the neighborhood level for specific countries or regions (Table 1). SEP indicators were used as categorical variables, coded as “low-medium-high”; “low” (the most deprived) was used as the reference in statistical analyses. SEP categories and cut points were specific to each cohort. Continuous indicators were categorized based on the tertile distribution. Additional information on the cohorts and SEP definitions is included in the Supporting Information. SEP category distributions are displayed in Supplementary Table S2.

### Statistical Analyses

We extracted exposure estimates for all exposome surfaces at participants’ home locations and conducted principal component analyses (PCAs) for each of the three domains of the external exposome as a dimension reduction technique to derive loadings for each cohort and each exposome domain. The domains were defined a priori to provide meaningful and interpretable characterization of the living environment for epidemiological studies investigating the health effects associated with different aspects of the external exposome. The distribution of the loadings for the first two principal components (PCs) across the different cohorts and geographical areas was used to interpret each PC. PCs are created based on the eigenvalues of the exposure correlation matrix and do not have any concrete meaning on their own. To ensure comparability and interpretability of the PCA results across cohorts and study sites, we applied a correction factor of −1 to the first PC of cohorts with negative loadings for a priori selected variables in each domain (NO_2_, impervious surface, and mean warm season temperature). As a result, countries with similar domain-specific exposure correlation structures are assigned harmonized exposome definitions. We conducted linear regression to estimate the association between SEP using “low” (the most deprived) as the reference category and the first PC of each external exposome domain. Separate analyses were conducted using individual- and area-level SEP indicators. All analyses were performed separately by cohort and adjusted for age if several age groups were present (adult and administrative cohorts). To understand the role of regional coverage and SEP indicators, we displayed the association between socioeconomic indicators and exposome domains’ PCs by geographical gradient (Northern vs Southern European countries), cohort type, and SEP indicator. To verify the possible impact of urbanicity gradients across cohort types, we conducted sensitivity analyses in the Catalan administrative cohorts and GCAT to (a) stratify and compare correlation structures and associations with SEP (area- and individual-level indicators) across urbanicity groups (urban, semiurban, and rural) in the Catalan administrative cohort and (b) compare findings in the GCAT cohort (mostly urban) and the analyses restricted to the urban part of the Catalan administrative cohort.

## Results

**Figure 2:**
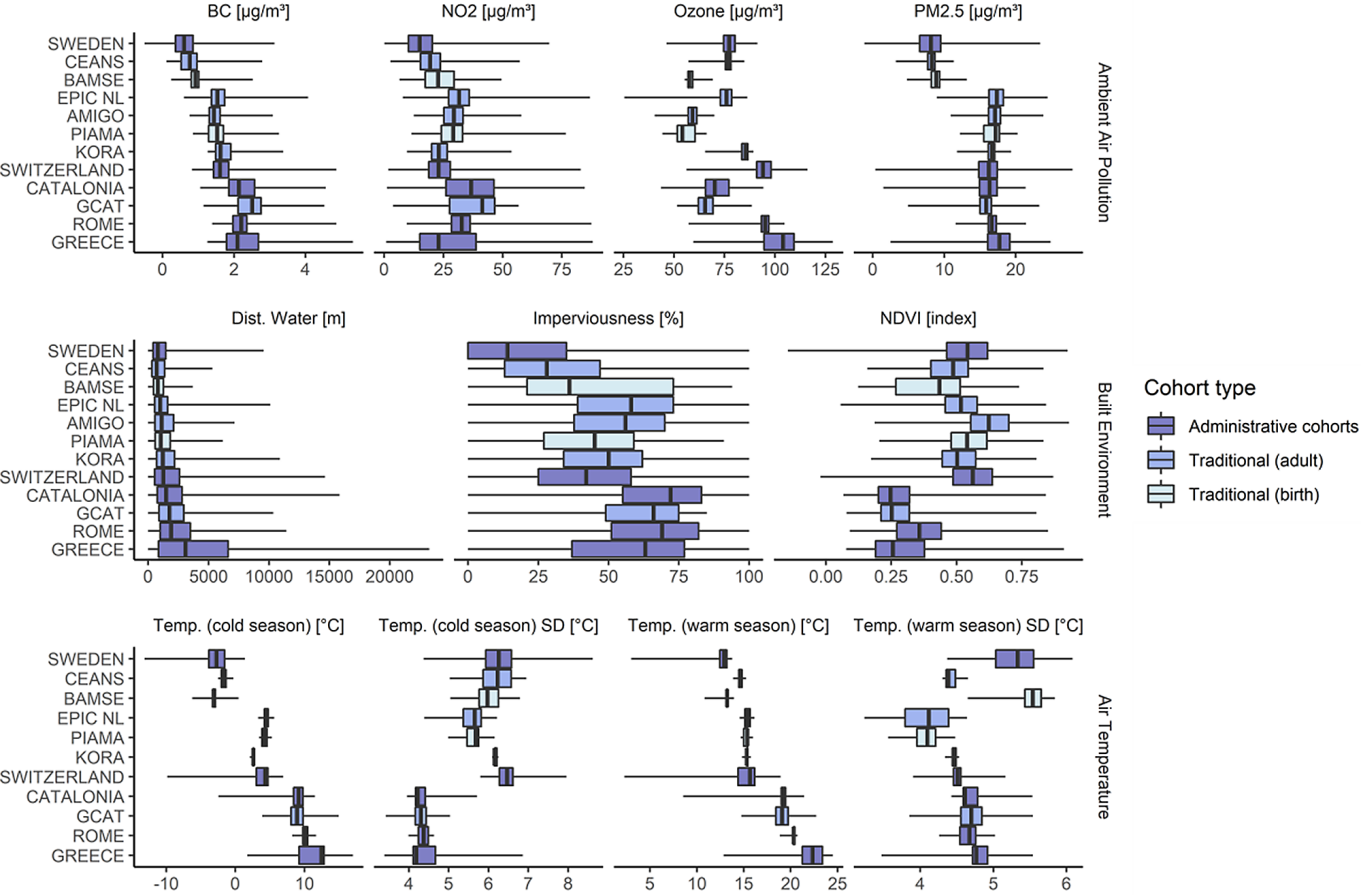
Exposure distribution by country and exposome domain. Cohorts are ordered from North (top) to South (bottom).

Figure 2 presents exposure distributions according to the cohort. Ambient air pollution was lowest in the three Swedish cohorts. NO_2_ and BC were highest in the Spanish, Greek, and Italian cohorts. Ozone concentrations were highest in the cohorts from Greece, Rome, and Switzerland. Cohorts from Northern Europe had higher NDVI and lower impervious surface levels compared to those in Southern Europe. Mean air temperature varied widely across cohorts, with higher temperatures observed in Southern European cohorts and larger variability (large IQRs) in the country-wide or regional cohorts compared to those focusing on one or few cities (e.g., Rome, CEANS, KORA, and EPIC-NL). Cold season temperature variability was smaller in the Southern compared to Northern cohorts. In contrast, summer temperature variability was highest in two of the three Swedish cohorts and lowest in two of the three Dutch cohorts. Exposome distributions by individual- and area-level SEP are displayed in Supplementary Table S3.

**Figure 3:**
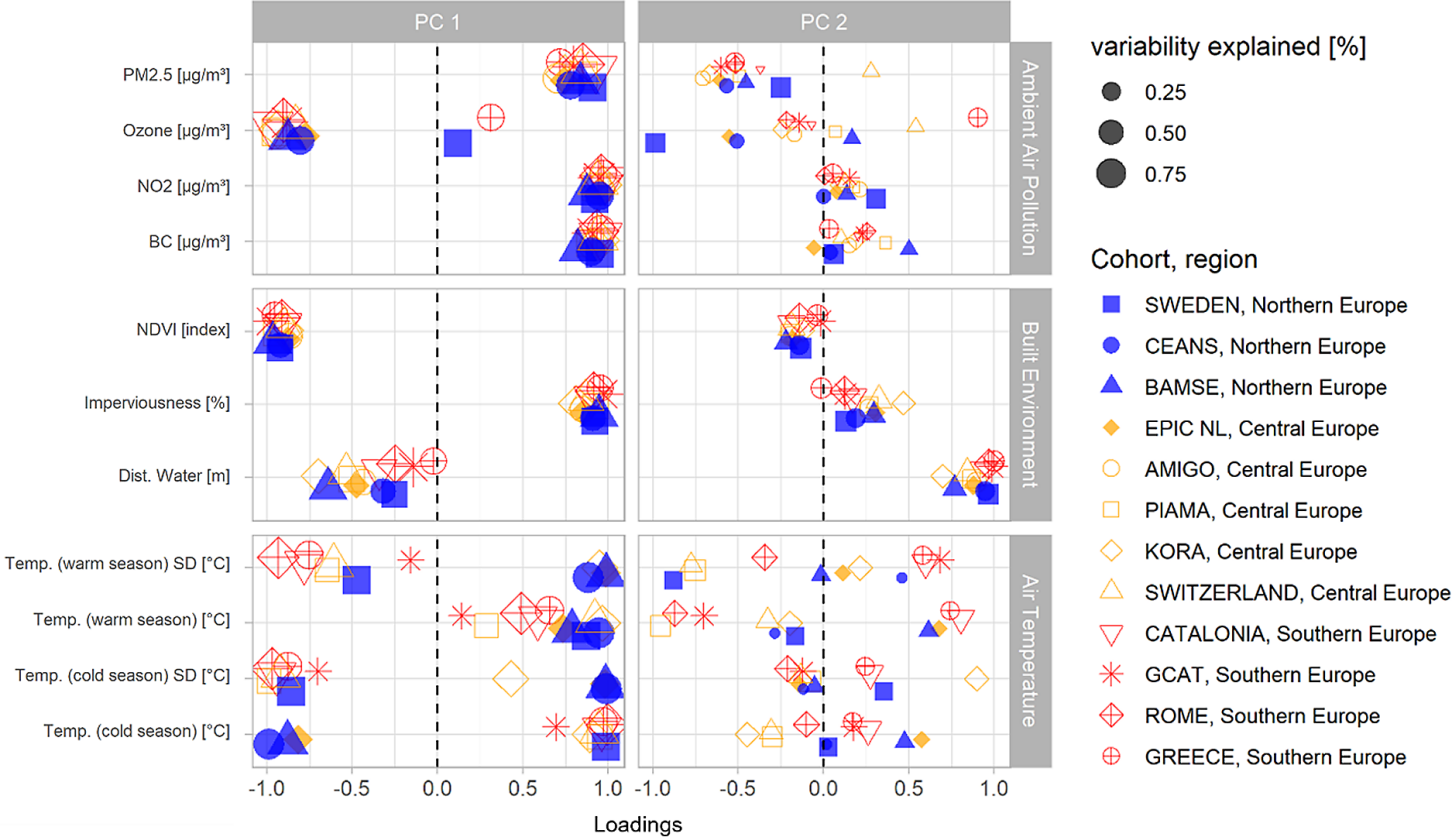
Distribution of PCA loadings by cohort and the external exposome domain. The symbol size is proportional to PC contribution of the exposure variables.

Distributions of the first and second PC loadings for the ambient air pollution and built environment domains were similar across most cohorts (Figure 3). For ambient air pollution, an increase in the first two PCs represents (1) increasing concentrations in traffic-related pollution (e.g., NO_2_, PM2.5, and BC) and (2) air pollution mixtures with lower concentrations of the particulate matter. In the Greek and Swedish administrative cohorts, where ozone was positively associated with traffic-related pollutants, an increase in the first PC represented higher concentrations of all the ambient air pollutants. The second PC for this domain was related to air pollution mixtures with lower proportions of BC compared to those of the other pollutants. In the built environment domain, the first two PCs can be interpreted as (1) increasing levels of the built surface (e.g., increased impervious surface and reduced NDVI) and (2) a larger distance to water bodies. For the air temperature domain, we observed different patterns across the cohorts. For most cohorts, the first PC represented a mixture of high temperature throughout the year and low seasonal standard deviation. Three cohorts (CEANS, BAMSE, and EPIC-NL) showed distinct patterns with higher temperature variability throughout the year and lower temperatures during the cold season. We did not identify any clear pattern in the second PC for the air temperature domain across cohorts (Figure 3). The variance explained by the first PCs is presented in Supplementary Table S4.

**Figure 4:**
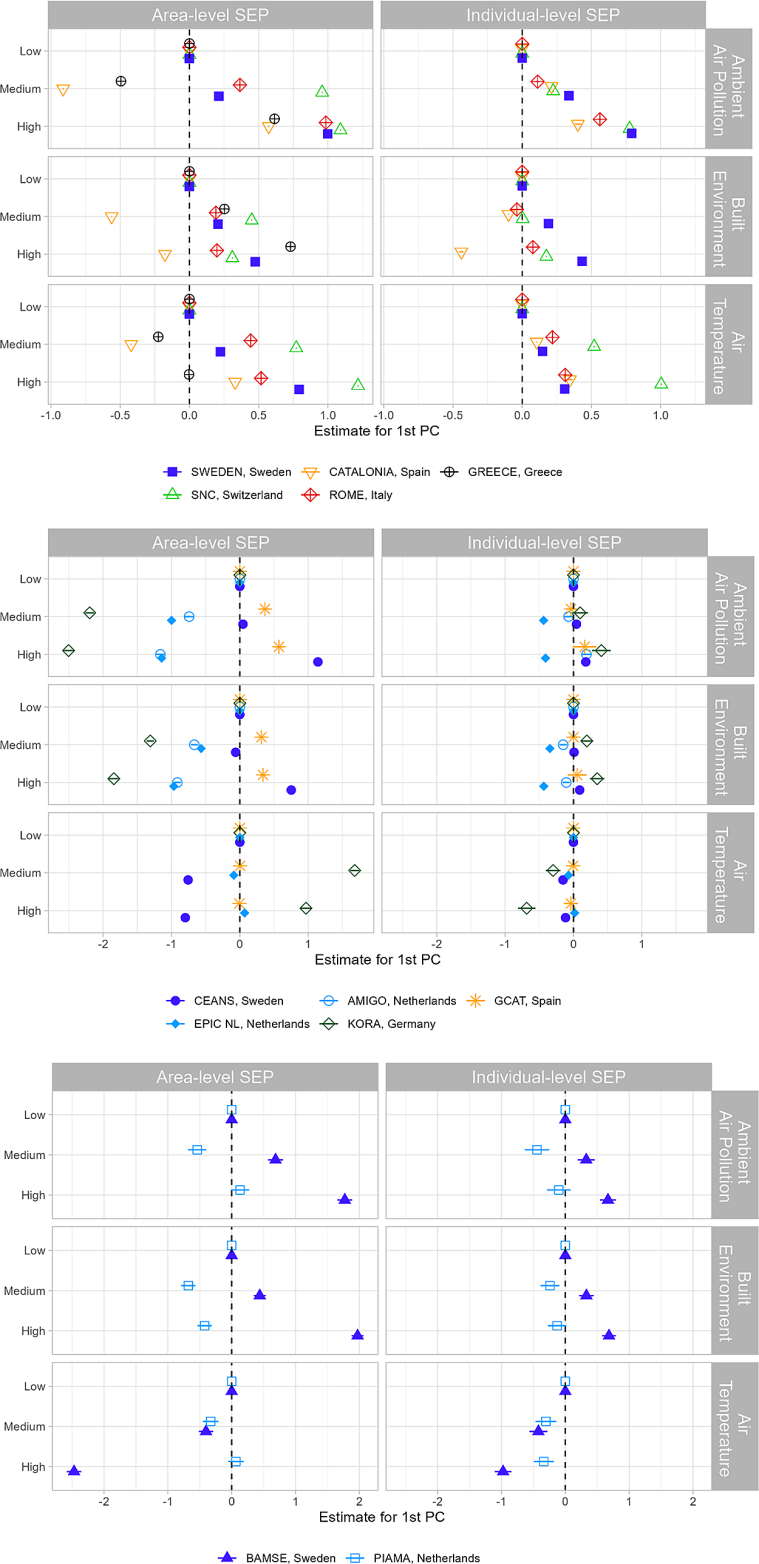
Association between the first PC of each domain of the external exposome and individual- and area-level SEP, by cohort type: administrative cohorts (top panel), adult cohorts (middle panel), and birth cohorts (bottom panel). Coefficient estimates are reported with 95% confidence intervals from the multivariable linear models adjusting for age (except birth cohorts). Estimates on the right side of the vertical dotted line represent a positive association between SEP and the first PC of each external exposome domain (e.g., higher levels of traffic-related pollution, built-up land use, and warm season mean temperature associated with higher SEP).

We found heterogeneous associations between the different domains of the external exposome and SEP across cohorts (Figure 4). The direction of the association between individual- and area-level indicators was mostly consistent within cohorts (except for KORA), and estimates were larger for area- compared to individual-level SEP. For most cohorts, participants living in areas with higher SEP had higher levels of traffic-related air pollution and built surface (more impervious surface and less green areas) compared to those living in low(er) SEP areas. In contrast, for the two Dutch cohorts and KORA, lower traffic-related air pollution and a built surface were associated with higher area-level SEP. The association between the first PC of the air temperature domain and SEP varied across cohorts and within countries, without a clear pattern. Associations between built surfaces and SEP were less consistent in terms of the direction for the two Catalan cohorts.

The most extreme associations between dimensions of the external exposome and area-level SEP were observed in traditional cohorts (Figure S1), specifically BAMSE and KORA. No clear pattern regarding the direction and strength of the association with the SEP indicator type was apparent (Figure S2). While administrative and traditional cohorts in the same region could be compared (Catalonia, Sweden), associations between traffic-related air pollution and area- and individual-level SEP were consistent across cohort types within the same region.

The results from the sensitivity analyses are presented in Supplementary Figures S3–S8. Domain-specific loadings were consistent across both Catalan cohorts and urbanicity groups. The stratified analyses in the Catalan administrative cohort showed mostly consistent estimates across urbanicity groups for the first two PCs of the ambient air pollution and built environment domains associated with SEP indicators. The coefficient size and direction differed across urbanicity for the temperature domain. The comparison between the urban portion of the Catalan administrative cohort and GCAT was mostly consistent for the ambient air pollution domain, but the results differed for the built environment in the first PC. The results were more consistent for the second PC. Supplementary Figures S9–S11 further present the results separately for cohort-specific area coverages (city, regional, and country-wide).

## Discussion

We characterized socioeconomic inequalities in multiple dimensions of the external exposome based on data from 25 million individuals participating in 12 European cohorts in seven different countries. Our analysis resulted in several key findings. First, PCA loadings reflecting multiple correlated exposures were broadly similar for traditional and administrative cohorts in the same country, indicating that the correlation structure within domains of the external exposome in the traditional cohorts was largely representative of the general population of the same country. Second, associations between the external exposome and SEP were stronger for area- compared to individual-level SEP indicators. Third, for most, but not all cohorts, higher exposure to traffic-related air pollution and built surfaces were associated with higher area-level SEP.

Overall, levels of environmental exposures and their correlations within the domain were consistent within each country, suggesting that geography is more relevant than the cohort type. Selected cohorts, such as birth or adult cohorts, showed similar exposure distributions to those of the administrative cohorts. For example, we found the lowest traffic-related pollution concentrations and impervious surface levels in the Stockholm area (Sweden) in agreement with previous evidence.^13−15^ We also observed a general north–south exposure gradient across all the exposome domains. For example, mean temperature and impervious surface were higher and the NDVI was lower in Greece, Italy, and Catalonia (Spain) compared to northern European countries. These findings suggest that traditional cohorts, which usually over-represent highly educated populations, present similar exposure profiles to those of administrative cohorts with reasonably good representation of the population at the national or regional level. Southern European countries also showed a particularly low standard deviation for the cold season temperature, while the highest values were observed in Sweden and Switzerland, reflecting the relatively stable winter climate in the Mediterranean region and the possible influence of cold episodes in colder countries.

These different climatic profiles were also reflected as differences in exposure correlation structures for the air temperature domain with varying distributions of temperature PC loadings across countries. Independent of regional differences in mean temperature, we could identify two groups of countries: those with low seasonal variability (most cohorts) and those with high temporal variability in both seasons (the three Swedish cohorts and EPIC-NL). The different patterns observed in EPIC-NL compared to the other two Dutch cohorts may be explained by larger day-to-day variations in certain regions of The Netherlands—possibly more rural—which are not reflected in the more uniform and urban traditional cohorts in this country. This interpretation is supported by the sensitivity analyses, which showed large differences in the association between the temperature domain and SEP indicators across urbanization groups in the Catalan administrative cohort. For the other two domains, we found a very similar distribution of exposure loadings across regions and cohort types.

Overall, the magnitude and direction of associations between the external exposome domains and SEP were heterogeneous. However, the most consistent patterns were (1) higher traffic-related pollution among individuals living in high-SEP areas and (2) stronger associations with area- compared to individual-level SEP. Our findings are in line with previous research showing wide variability in the association between SEP and urban environmental exposures in the European region,^3,16^ where some urban centers can have high property values but also high traffic volume and air and noise pollution. In this regard, the European context contrasts with other regions such as North America, where air pollution, lack of green space, and poor walkability are consistently more frequent in socioeconomically deprived areas.^16,17^ The heterogeneity of associations across cohorts and countries can be related to the large geographical areas included in our study, including urban centers and larger regional/national cohorts. It can also be leveraged in epidemiological studies to examine various confounder structures when investigating socioeconomic determinants of health. As our aim was to characterize the external exposome across regions and SEP using an agnostic approach, we did not adjust for urbanicity or area-level administrative units. Nevertheless, the associations remained mostly consistent within countries (e.g., Sweden and The Netherlands, only partially in Spain) and across urbanization grades (sensitivity analyses in Catalonia), suggesting large-scale differences in SEP–exposome inequalities. Besides, regional differences such as land use and other built-up characteristics, as well as differences in individual interaction with the living environment, may not be captured by these exposome indicators. Stronger associations of environmental exposures at residence with area- compared to individual-level SEP have been reported previously^3,18^ and are likely due to the more consistent spatial patterning of area-level SEP. Furthermore, area-level SEP indicators are of particular interest because they give indications of clustering of poverty with other types of environmental disadvantages.^19^ Different conceptual approaches are usually applied for area- and individual-level indicators because of their independent action on health.^5,19^ While the association between exposome characteristics and individual-level SEP reported in our study has been described before,^3,5^ neighborhood deprivation is of particular interest for public health interventions because they can be acted upon with possible large-scale health impacts.^5^

The diversity of SEP indicators and cut points used in the categorization of socioeconomic variables may also influence our findings. In our study, the two Spanish cohorts and the Swedish national cohort presented very low proportions of high SEP for the individual-level indicator, making it difficult to identify specific trends according to the individual-level SEP. A recent study investigating the socioeconomic disparity in the health impacts of green space and air pollution found large geographical variability in the geographical distribution of the deprived population across various European cities,^20^ which can explain the regional differences in the association between deprivation and suboptimal environmental factors observed in our study. Recent evidence also suggests that higher SEP may be associated with increased external contaminants but a healthier lifestyle and diet, suggesting that regional disparities may be counterbalanced by other individual and behavioral determinants of health.^6^ Differences in the temporal availability of exposome variables and SEP indicators as well as possible exposure misclassification may further affect the findings.

PCA within specific exposure domains is a valuable tool for epidemiological studies involving multiple exposures in multicountry analyses. This approach has been successfully applied in a previous study involving three adult and four administrative cohorts from the EXPANSE consortium focusing on the external exposome and stroke incidence.^2^ In the present study, we identified interpretable PCs for ambient air pollution and built environment domains but not for air temperature. The correlation structure of air temperature in different seasons and its temporal variability within the cohort is a function of many factors including land use, climate, latitude, and geographical coverage of the cohort. Alternative indicators may be more relevant to the urban exposome such as those capturing the urban heat-island effect.

This is one of the first studies to systematically characterize correlation structures of multiple domains of the external exposome and their association with SEP using an exposome framework.^3,5,6^ Our study included a large sample of more than 25 million individuals, representative of many European countries. Including colocated administrative and traditional cohorts enabled us to gain insight into the influence of geographic differences and cohort selection effects on socioeconomic inequalities in the external exposome. However, several limitations should be considered. We had a limited number of exposures included within each domain, and future work should include a wider range of environmental exposures harmonized at the European level. The lack of harmonized socioeconomic indicators, as well as their different cut points and spatial resolution, made direct comparisons of socioeconomic inequalities in the external exposome across cohorts challenging. Our analysis considered only exposures in the surrounding residential environment, potentially capturing mechanisms related to how SEP influences where individuals are able or choose to live.^1^ We did not have consistent data in all cohorts on ethnicity, health behaviors, or psychosocial exposures (e.g., stress and social network) which reflect other pathways by which SEP can influence the exposome. Furthermore, the limited number of colocated studies and their diversity in cohort types and spatial extents limit our ability to identify the exact source of variability in the association between the exposome and SEP. Our sensitivity analyses suggest that urban–rural gradients are important drivers in geographical differences observed for the temperature domain and possibly some aspects of the built environment. In contrast, the association between traffic-related pollution and SEP indicators was robust across environments with different urbanicity levels. While vast improvements have been made in generating harmonized data to characterize the physicochemical environment across Europe,^15^ similar developments to generate harmonized area- and individual-level socioeconomic indicators are still lacking, presenting a bottleneck for exposome research.^1^

Leveraging large data sets representing a wide range of European regions for the EXPANSE project, we provide new evidence indicating that external exposome correlation structures were consistent across cohorts in the same regions, making it possible to derive interpretable, domain-specific exposome definitions across countries. However, patterns of SEP inequalities in the external exposome were heterogeneous in magnitude and direction, indicating that the role of SEP as a coexposure or confounder of other external exposome domains is highly context-dependent. This heterogeneity represents an opportunity for epidemiological studies investigating the impact of SEP on health and how they modulate the environment and health associations. More detailed and harmonized SEP indicators are needed for future research.

## Supporting Information Available

Description of the cohorts included in the study; additional information on the exposure data source used for exposome assessment, including source and time period; overview of the source, definition, and distribution of the SEP variables in each cohort; exposure distribution by individual- and area-level SEP categories; from the PCA analyses, overview of the variance explained by the first PCs, by cohort and domain; visual overview of the association between the first PC of each domain of the external exposome and individual- and area-level SEP, stratified by cohort type and SEP indicator; visual overview of the distribution of PCA loadings by cohort and external exposome domain in the Catalan administrative cohort, stratified by urbanicity groups, and separately for the GCAT cohort and the urban portion of the Catalan administrative cohort; visual overview of the association between the first and second PCs of each domain of the external exposome and individual- and area-level SEP in the Catalan administrative cohort, stratified by urbanicity group, and separately for the GCAT cohort and the urban subset of the Catalan administrative cohort; and association between the first PC of each domain of the external exposome and individual- and area-level SEP, stratified by city geographic coverage (city or municipality, regional, and country-wide) (PDF)
