# Supplementary material for: Socioeconomic Inequalities in the External Exposome in European Cohorts: The EXPANSE Project
Source: Environ Sci Technol. 2024 Sep 5;58(37):16248–57. doi: 10.1021/acs.est.4c01509 (PMC11411702; doi:10.1021/acs.est.4c01509)
Supplement: Supplementary file 1 — es4c01509_si_001.pdf [file es4c01509_si_001.pdf]

Supporting information

Manuscript: Socioeconomic inequalities in the external exposome in European cohorts: the EXPANSE project

Authors: Apolline Saucy[1,2,3*], Fabián Coloma[1,2,3], Sergio Olmos[1,2,3], Christofer Åström[4], Natalia Blay[5], Jolanda M.A. Boer[6], Payam Dadvand[1,2,3], Jeroen de Bont[7], Rafael de Cid[5], Kees de Hoogh[8,9], Konstantina Dimakopoulou[10], Ulrike Gehring[11], Anke Huss[11], Dorina Ibi[11], Klea Katsouyanni[10,12], Gerard Koppelman[13, 14], Petter Ljungman[7,15], Erik Melén[16,17], Mark Nieuwenhuijsen[1,2,3], Federica Nobile[7,18], Annette Peters[19,20], Regina Pickford[19], Roel Vermeulen[11], Danielle Vienneau[8,9], Jelle Vlaanderen[11], Kathrin Wolf[19], Zhebin Yu[7], Evangelia Samoli[10†], Massimo Stafoggia[7,18†], Cathryn Tonne[1,2,3†*], *on behalf of the EXPANSE Project Team*

[†]Equal contribution

* Corresponding authors

[1] ISGlobal, Barcelona, Spain

[2] Universitat Pompeu Fabra (UPF), 08003, Barcelona, Spain

[3] CIBER Epidemiología y Salud Pública (CIBERESP), 28029, Madrid, Spain

[4] Department of Public Health and Clinical Medicine, 901 87, Umeå University, Umeå, Sweden

[5] Genomes for Life-GCAT Lab, German Trias i Pujol Research Institute (IGTP), 08916, Badalona, Spain

[6] National Institute for Public Health and the Environment, 3721, Bilthoven, the Netherlands

[7] Institute of Environmental Medicine, Karolinska Institutet, 171 77, Stockholm, Sweden

[8] Swiss Tropical and Public Health Institute Basel, 4123, Allschwil, Switzerland

[9] University of Basel, 4001, Basel, Switzerland

[10] Department of Hygiene, Epidemiology and Medical Statistics, Medical School, National and Kapodistrian University of Athens, 115 27, Athens, Greece

[11] Institute for Risk Assessment Sciences (IRAS), Utrecht University, 3584, Utrecht, The Netherlands

[12] MRC Centre for Environment and Health, School of Public Health, Imperial College London, W2 1PG, London, UK

[13] Department of Pediatric Pulmonology, Beatrix Children's Hospital, University Medical Center Groningen, University of Groningen, 9713, Groningen, The Netherlands

[14] Groningen Research Institute for Asthma and COPD, University of Groningen, 9713, Groningen, The Netherlands

[15] Department of Cardiology, Danderyd Hospital, 171 77, Stockholm, Sweden

[16] Department of Clinical Sciences and Education, Södersjukhuset, Karolinska Institutet, 171 77, Stockholm, Sweden

[17] Sachś Children and Youth Hospital, Södersjukhuset, 118 61, Stockholm, Sweden

[18] Department of Epidemiology, Lazio Region Health Service / ASL Roma 1, 00147, Rome, Italy

[19] Institute of Epidemiology, Helmholtz Zentrum München, German Research Center for Environmental Health, 85764, Neuherberg, Germany

[20] IBE, Faculty of Medicine, Ludwig-Maximilians-Universität, 81377, Munich, Germany

Summary: 23 pages, 11 figures, 4 tables.

Supplementary information

Cohorts and SEP variables description:

The **CEANS (Cardiovascular Effects of Air Pollution and Noise in Stockholm)** is composed of four subcohorts: The Screening Across the Lifespan Twin Study (SALT) sampled 7,043 individuals from the Swedish Twin Register born 1958 and earlier, who lived in Stockholm County [1]. The Stockholm Diabetes Preventive Program (SDPP) is a population-based prospective study of 7,949 subjects aged 35–54 years [2]. The SIXTY subcohort consists of a random population sample of one-third of all men and women living in Stockholm County turning 60 years between August 1997 and March 1999 [3]. Lastly, The Swedish National Study of Aging and Care in Kungsholmen (SNAC-K) randomly sampled individuals 60+ years of age from a central area in Stockholm [4]. All participants resided in Stockholm County, Sweden. Individual SEP data was self-reported highest education, collected at baseline questionnaire. Area-SEP was median income at the "Small Area Market Statistics" (SAMS) unit for 2001.

The **SIMSAM** is a register-based cohort with all adults in Sweden. For the study all individuals aged above 37 in 2010 was included. The cohort contain data on all individuals up until exclusion (incident, death, migration etc.) or the end of the follow-up period. Individual-level (age, sex, living condition, education, etc.) and area-level (income, education, etc.) information, through geocoded residential addresses for each year, is available for each subject. Area-level income was averaged over the geographical unit called district, defined by Statistics Sweden.

The **Swiss National Cohort (SNC)** is a nation-wide cohort linking census data with birth, mortality, and emigration data [5,6]. In 2010, Switzerland replaced the classic door-to-door census with the registry-based census repeated each year. In this new structure, data on education, occupation and employment is only collected in an annual structural enquiry of a random sample of about 250,000 people per year. Thus, due to incompleteness in these individual-level variables, we used the available Swiss-SEP index [7,8] developed within the framework of the SNC. The index, calculated for small neighbourhoods of the nearest 50 households, was constructed on median rent, crowding, and proportion households headed by a low educated person and/or by a person in unskilled occupation. We used the original Swiss-SEP to represent the individual-SEP variable, and aggregated the values to community level to derive the area-SEP variable for this analysis.

EPIC-NL (European Prospective Investigation into Cancer and Nutrition, the Netherlands) combines two EPIC-cohorts within the Netherlands: The Monitoring Project on Risk Factors and chronic diseases in the Netherlands (MORGEN) cohort which consists of a general population sample aged 20–59 years from three Dutch towns (Amsterdam, Doetinchem and Maastricht). Prospect is a prospective cohort study among women aged 49–70, residing in the city of Utrecht or its vicinity, who participated in the nation-wide Dutch breast cancer screening programme between 1993 and 1997 [9]. The highest obtained educational level was used as a measure of individual SES. Educational level was self-reported in the general questionnaire at baseline. The cohort was divided into three groups: low educational level (attended primary school only), medium educational level (attended secondary school or lower and intermediate vocational education) and high educational level (higher vocational education or university). Area-level SES data were at the neighborhood-level ("Buurt") from Statistics Netherlands [10]. In the Netherlands, neighborhoods are defined as "part of a municipality dominated by a given type of land use or buildings. For EPIC_NL, the percentage low income households was used as a proxy for the area-level SES. The percentage is stated for at least 70 private households per neighbourhood.

AMIGO is a sample of 14,829 working-age adults in the Netherlands selected between 31 and 65 years of age at baseline. It is a representative sample of the adult population of working age in the Netherlands created to investigate occupation and environmental health from a 'multidisciplinary and life-course perspective [11]. Recruitment happened between 2011 and 2012, where individual, socioeconomic, behavioural and health characteristics were collected. Individual SEP data was self-reported highest completed education, collected at baseline. Area-level SEP data were at the neighborhood-level ("Buurt") from Statistics Netherlands [10]. In the Netherlands, neighborhoods are defined as "part of a municipality dominated by a given type of land use or buildings.

The Rome Longitudinal Study (RoLS) includes adults aged 30 years and older who were resident in Rome on October 9th, 2011 (the reference day of the Census). The population-based cohort was followed-up until 2018 and the subjects were linked to Health Information Systems available in the Lazio Region through an anonymous identification code. Individual-level (age, sex, marital status, occupation status, education, etc.) and area-level (deprivation index, unemployment rate, etc.) information, through geocoded residential addresses at baseline, is available for each subject. The enrolment and selecting procedures were similar to those described in more detail for the 2001 RoLS [12,13]. Individual SEP was self-reported educational level (low = primary school + middle school; middle = high school; high = university or more), collected from the 2011 National Census data. Area-level SEP was a reversed deprivation index (tertiles) at census block (n = 13,099), calculated through a factor analysis from 2001 Census data on several socioeconomic parameters (occupation, education, housing tenure, family composition, and foreign status) [14].

PIAMA is a birth cohort from the Netherlands, that was set up to investigate the effect of mite-allergen avoidance on the incidence of childhood asthma and allergy and to assess lifestyle and environmental risk factors for childhood asthma and allergy [15]. Individual SEP data was self-reported highest parental education, collected at baseline questionnaire. Area-level SEP data were at the neighborhood-level ("Buurt") from Statistics Netherlands [10]. In the Netherlands, neighborhoods are defined as "part of a municipality dominated by a given type of land use or buildings.

BAMSE is an ongoing longitudinal, population-based prospective birth cohort including 4,089 children born between 1994 and 1996 in Stockholm, Sweden, designed to study risk factors for asthma, allergic diseases and lung function in childhood. Individual SEP data was self-reported highest parental education, collected at baseline questionnaire. Area-level SEP was median income at the "Small Area Market Statistics" (SAMS) unit, which refer to "the smallest areal units in a system of geographical co-ordinates areas in Stockholm and 9,281 SAMS areas in the rest of Sweden" [16].

The Greek administrative cohort (GRAD), is a nationwide population-based cohort. GRAD includes all adults with a social security number aged 37+years in 2014 and follows them until the end of 2019. The cohort contains data on disease diagnoses and related medication subscription and is linked with the mortality registry of the Hellenic Statistical Authority. The administrative cohort includes Individual-level (age, sex, education) information and is linked to small area-level (income, education, unemployment etc.) data from the census 20011 linked to the geocoded residential addresses. Area-level SEP variables for the Greater Area of Athens and other large municipalities (population greater than 100,000 inhabitants) in Greece, were available at square-block level. For the rest of the areas in Greece, the variables were available at municipality unit level. The study was approved by the

[1] https://www.idika.gr/

Committee of Ethics and Research Ethics, National and Kapodistrian University of Athens and by the Bioethics Committee of Medical School, National and Kapodistrian University of Athens.

KORA cohort (Cooperative Health Research in the Region of Augsburg) is a regional research platform for population-based surveys and subsequent follow-up studies in the fields of epidemiology, health economics, and health care research [17,18]. This analysis includes two cross-sectional population-representative surveys that were conducted in 1994-1995 (S3 survey) and 1999-2001 (survey S4) in the city of Augsburg and two adjacent rural districts including inhabitants of German nationality aged 25 to 74. Individual SEP was self-reported educational level (low = primary school or less; middle = up to secondary school or equivalent; high = university degree or more). Area-level SEP was the median household Income (low: < 1.750 €; middle: 1.750 - 2.250 €; high: >=2.250 €) on a 5km * 5km raster assigned to the residential addresses.

The Catalonia administrative cohort is a population-based cohort of the adult population of Catalonia (the northeast region of Spain), built through record linkage using data collected in the public health administration databases of Catalonia for the COVAIR-CAT study. The cohort includes adults (≥18 years) residents of Catalonia who were covered by the public healthcare system in 2015, i.e. nearly the entire Catalan population (98.8% in 2015). Catalonia is composed of 947 municipalities grouped in seven health regions (median area of 5425 km^2) [19]. Individual SEP data was income collected from the Central Registry of Insured Persons and was based on the co-payment system for drug dispensations. Area-level SEP was the socioeconomic index (PSCA) available at primary care service area level. This index concentrates information on employment status, educational level, immigration and income of all the people residing in each territorial unit, based on 6 sectoral indicators [20].

GCAT (Genomes for life | Cohort Study of the genomes of Catalonia) is an ongoing longitudinal, prospective adult population-based cohort of 20.000 people from Catalonia (Spain), recruited in Catalonia at 2014-2017 [21]. GCAT was set up to facilitate the prediction, prevention and personalized treatment of complex and chronic diseases such as diabetes, heart disease, respiratory diseases and cancer, and comprehensively investigate the complex interplay among genetics, lifestyle and environmental factors that influence the risk of developing the disease and impact its treatment. GCAT includes people from Catalonia of 40-65 years old at recruitment, linking Electronic-Health Records from the National Health Public System and genomic information. Individual SEP data was self-reported income, collected at baseline questionnaire. Area-level SEP data were deprivation index at enumeration district-level from 2011.

Supplementary tables

Supplementary Table S1. Data source, temporality and time period of the exposure assessment.

Exposome domain	Exposure variables	Source	Temporal resolution	Year
**Ambient air pollution**	$PM_{2.5}$, NO_2, BC, O_3	ELAPSE study (Land use regression models), 100mx100m	Annual	2010
**Land/Built environment**	NDVI	Terra Moderate Resolution Imaging Spectroradiometer (MODIS) with 250 m x 250 m	Annual	2019
	Impervious surface	Copernicus Land Monitoring Service within 100mx100m grid cell	Annual	2015
	Distance to blue space	EU-Hydro map (Copernicus Land Monitoring Service)	-	-
**Ambient temperature**	Seasonal mean temperature	European Centre for Medium-Range Weather Forecasts (ECMWF) ERA5-Land	Warm and cold season	2010
	Seasonal temperature standard deviation		Warm and cold season	2010

Supplementary Table S2: Overview of the source, definition and distribution of the SEP variables in each cohort. The %SEP refer to the percentage of the cohort belonging to each of the 3 SEP categories operationalized either by cutpoints defined by the cohorts or tertiles of continuous variables.

Cohort	Individual SEP variable	Variable grouping	%Low %Med. %High	Area-level SEP variable	Variable grouping	%Low %Med. %High
Switzerland (SNC)	Swiss index	Tertiles	33.1<br>33.3<br>33.6	Swiss index, mean at community level (n= 2583)	Tertiles	34.5<br>34.2<br>31.3
ROME	Education	Cohort's definition	40.2<br>37.1<br>22.7	Reverse deprivation index at census block level	Tertiles	33.3<br>33.3<br>33.3
GREECE	-	-	-	% tertiary education at municipality level or square block level for cities with population > 100,000,	Tertiles	32.8<br>32.8<br>34.4
SWEDEN (SIMSAM)	Education	Cohort's definition	71.0<br>27.9<br>1.1	Mean income at district level	Tertiles	33.3<br>33.3<br>33.3
CATALONIA	Income	Tertiles	70.8<br>28.6<br>0.6	Socioeconomic index (PSCA) at primary care service area level	Tertiles	33.3<br>33.3<br>33.3
EPIC-NL	Education	Cohort's definition	33.6<br>33.4<br>33.1	Inverse % of low income at neighborhood level	Tertiles	33.3<br>33.3<br>33.3
AMIGO	Education	Cohort's definition	31.0<br>31.4<br>37.6	Inverse % low income at neighborhood level ("Buurt")	Tertiles	30.5<br>31.9<br>37.6
GCAT	Family incom e	Tertiles	15.9<br>81.4<br>2.7	Inverse deprivation index at census district level	Tertiles	33.3<br>33.3<br>33.3
KORA	Education	Cohort's definition	12.7<br>68.0<br>19.3	Mean income	Tertiles	43.4<br>39.9<br>16.7
CEANS	Education	Cohort's definition	31.3<br>36.9<br>31.8	Mean income at Small Area Market Statistics level (SAMS)	Tertiles	33.7<br>33.7<br>32.7
BAMSE	Household SEP	Tertiles	33.6<br>28.9<br>37.5	Median income at SAMS	Tertiles	33.5<br>33.2<br>33.3
PIAMA	Parental Education	Cohort's definition	12.7<br>36.7<br>50.6	SEP score at neighborhood level ("Buurt")	Tertiles	31.9<br>33.7<br>34.4

Supplementary Table S3: Exposure distribution by individual SEP categories.

cohort	SEP (individual)	Quantile distribution	NO2 [µg/m^3]	PM2.5 [µg/m^3]	Black carbon [µg/m^3]	Ozone [µg/m^3]	NDVI [index]	Imperviousness [%]	Dist. Water [m]	Temp. (warm season) [°C]	Temp. (warm season) SD [°C]	Temp. (cold season) [°C]	Temp. (cold season) SD [°C]
	low	0%	12,9	12,0	0,8	40,4	0,2	0,0	0,0	8,3	5,9	8,3	5,9
	low	25%	25,0	16,3	1,3	57,3	0,6	41,0	583,1	8,8	7,3	8,8	7,3
	low	50%	29,1	17,2	1,4	59,3	0,6	59,0	1081,7	9,1	7,7	9,1	7,7
	low	75%	32,9	18,0	1,6	61,3	0,7	72,0	2126,0	9,2	7,8	9,2	7,8
	low	100%	56,1	22,9	2,8	70,0	0,9	100,0	6879,7	9,6	8,0	9,6	8,0
	middle	0%	12,5	12,0	0,8	45,2	0,3	0,0	0,0	8,3	5,9	8,3	5,9
	middle	25%	25,0	16,1	1,3	57,3	0,6	37,0	583,1	8,8	7,4	8,8	7,4
**AMIGO**	middle	50%	28,9	17,0	1,4	59,3	0,6	56,0	1131,4	9,0	7,7	9,0	7,7
	middle	75%	32,9	17,8	1,6	61,4	0,7	70,0	2193,2	9,2	7,8	9,2	7,8
	middle	100%	58,0	23,9	3,1	70,0	0,9	100,0	7158,9	9,6	8,0	9,6	8,0
	high	0%	13,0	10,9	0,8	44,7	0,2	0,0	0,0	8,3	5,9	8,3	5,9
	high	25%	25,8	16,2	1,3	56,8	0,6	35,0	565,7	8,8	7,4	8,8	7,4
	high	50%	30,0	17,0	1,5	58,7	0,6	54,0	1104,5	9,0	7,6	9,0	7,6
	high	75%	34,0	17,8	1,6	61,1	0,7	69,0	2002,5	9,2	7,8	9,2	7,8
	high	100%	55,2	22,8	2,9	70,0	0,9	100,0	7083,8	9,6	8,0	9,6	8,0
	low	0%	10,6	5,6	0,3	55,3	0,1	0,0	0,0	10,8	4,9	-6,2	5,3
	low	25%	16,5	7,9	0,8	57,0	0,4	20,0	500,0	13,2	5,5	-3,3	6,0
	low	50%	19,6	8,8	0,9	57,8	0,5	30,0	905,5	13,2	5,5	-3,1	6,0
	low	75%	26,7	9,3	1,0	59,9	0,5	48,0	1426,5	13,3	5,7	-3,1	6,3
	low	100%	36,4	13,1	1,7	66,3	0,7	93,0	3601,4	13,4	5,8	-0,8	6,4
	middle	0%	6,4	4,8	0,3	55,8	0,1	0,0	100,0	10,9	4,7	-6,0	5,5
	middle	25%	16,9	8,2	0,8	57,0	0,3	20,0	500,0	13,1	5,4	-3,3	5,8
**BAMSE**	middle	50%	22,8	8,9	0,9	57,6	0,4	35,0	806,2	13,2	5,5	-3,1	6,0
	middle	75%	29,6	9,3	1,0	59,4	0,5	73,0	1315,3	13,3	5,7	-2,9	6,3
	middle	100%	43,1	10,6	2,1	67,0	0,7	94,0	3687,8	13,4	5,7	-2,7	6,7
	high	0%	6,8	5,3	0,2	55,5	0,1	0,0	0,0	12,8	4,6	-3,8	5,0
	high	25%	18,6	8,3	0,8	56,8	0,2	22,0	400,0	13,1	5,4	-3,1	5,8
	high	50%	26,4	8,9	0,9	57,5	0,4	43,0	707,1	13,2	5,5	-3,1	6,0
	high	75%	30,6	9,3	1,0	58,7	0,5	78,0	1081,7	13,2	5,5	-2,9	6,0
	high	100%	49,5	13,1	2,5	69,2	0,7	94,0	3687,8	14,0	5,8	0,4	6,8
	low	0%	1,2	1,5	1,1	43,5	0,1	0,0	0,0	8,5	4,4	-2,5	4,0
	low	25%	25,5	14,9	1,8	65,8	0,2	56,0	721,1	19,0	4,6	8,4	4,2
	low	50%	36,0	16,3	2,1	70,5	0,2	73,0	1456,0	19,1	4,6	9,1	4,2
	low	75%	46,2	17,3	2,6	77,5	0,3	83,0	2778,5	19,6	4,8	9,7	4,4
	low	100%	84,7	21,2	4,6	94,3	0,8	100,0	15842,0	21,5	5,5	11,5	5,7
	middle	0%	1,6	2,5	1,1	43,5	0,1	0,0	0,0	8,5	4,4	-2,5	4,0
	middle	25%	27,5	15,1	1,9	65,1	0,2	53,0	800,0	19,0	4,6	8,5	4,1
**CATALONIA**	middle	50%	38,2	16,4	2,2	69,6	0,2	71,0	1562,0	19,1	4,6	9,1	4,2
	middle	75%	46,6	17,5	2,6	76,0	0,3	82,0	2865,3	19,4	4,8	9,7	4,4
	middle	100%	84,7	21,4	4,6	93,9	0,8	100,0	15572,4	21,5	5,5	11,5	5,7
	high	0%	3,0	4,4	1,1	52,9	0,1	0,0	0,0	8,5	4,4	-2,4	4,0
	high	25%	28,6	15,1	1,9	64,7	0,2	41,0	860,2	19,0	4,6	9,1	4,1
	high	50%	41,2	16,6	2,3	68,3	0,3	65,0	1824,8	19,2	4,6	9,4	4,2
	high	75%	46,7	17,4	2,8	75,2	0,4	79,0	2906,9	19,3	4,7	9,7	4,3
	high	100%	70,8	20,8	3,7	92,9	0,8	100,0	12478,8	21,5	5,5	11,5	5,7
	low	0%	2,7	3,4	0,1	58,2	0,2	0,0	0,0	13,9	4,3	-2,5	5,0
**CEANS**	low	25%	15,3	7,7	0,5	75,6	0,4	14,0	316,2	14,5	4,4	-2,1	5,9
	low	50%	19,2	8,2	0,7	76,9	0,5	29,0	806,2	14,7	4,4	-1,6	6,2

	low	75%	23,3	8,7	1,0	78,2	0,5	47,0	1476,5	14,8	4,5	-1,4	6,6
	low	100%	57,2	11,0	2,8	83,8	0,8	100,0	4701,1	15,3	4,7	-0,3	6,9
	middle	0%	2,9	3,2	0,1	57,2	0,2	0,0	0,0	13,9	4,3	-2,4	5,0
	middle	25%	15,2	7,7	0,5	75,5	0,4	12,0	300,0	14,5	4,4	-2,0	5,8
	middle	50%	19,1	8,2	0,8	77,0	0,5	28,0	700,0	14,7	4,4	-1,6	6,2
	middle	75%	23,4	8,7	1,0	78,3	0,5	47,0	1392,8	14,8	4,5	-1,2	6,6
	middle	100%	47,3	11,3	2,1	84,9	0,8	100,0	4904,1	15,3	4,7	-0,3	6,9
	high	0%	3,0	3,6	0,2	59,9	0,2	0,0	0,0	13,9	4,3	-2,4	5,0
	high	25%	15,7	7,7	0,6	75,4	0,4	12,0	223,6	14,6	4,4	-2,0	6,0
	high	50%	19,9	8,2	0,8	76,8	0,5	27,0	583,1	14,7	4,4	-1,6	6,2
	high	75%	24,7	8,7	1,0	78,1	0,5	48,0	1204,2	14,8	4,5	-1,5	6,6
	high	100%	48,4	11,1	2,2	83,6	0,8	100,0	5315,1	15,3	4,6	-0,3	6,9
	low	0%	8,3	8,0	1,4	53,3	0,1	0,0	0,0	15,3	4,0	4,4	3,6
	low	25%	28,2	15,0	2,1	62,1	0,2	50,0	900,0	18,4	4,5	8,1	4,2
	low	50%	41,7	15,9	2,5	65,3	0,3	66,0	1655,3	19,1	4,7	8,9	4,3
	low	75%	46,3	16,7	2,7	69,2	0,3	75,0	2860,1	19,8	4,8	9,8	4,4
	low	100%	55,3	23,3	4,2	84,1	0,7	85,0	9740,6	22,6	5,5	15,0	4,9
	middle	0%	3,8	4,9	1,1	51,5	0,1	0,0	0,0	14,7	3,9	3,9	3,4
	middle	25%	27,3	15,0	2,1	62,1	0,2	49,0	894,4	18,4	4,6	8,0	4,2
**GCAT**	middle	50%	41,3	15,9	2,5	65,3	0,3	66,0	1749,3	19,1	4,7	8,9	4,3
	middle	75%	46,7	16,6	2,8	69,4	0,3	75,0	2961,4	19,7	4,8	9,8	4,4
	middle	100%	56,8	22,2	4,5	88,5	0,8	85,0	10358,6	22,7	5,5	14,0	5,0
	high	0%	12,9	10,4	1,5	54,9	0,2	0,0	0,0	16,0	3,9	4,4	3,8
	high	25%	31,8	15,1	2,2	61,7	0,2	48,0	905,5	18,4	4,6	8,0	4,2
	high	50%	42,1	15,8	2,6	64,5	0,2	67,0	2121,3	19,1	4,7	8,9	4,3
	high	75%	47,8	16,4	2,8	69,3	0,3	76,0	3157,5	19,7	4,8	9,8	4,4
	high	100%	54,0	19,6	3,2	83,1	0,7	84,0	7700,6	21,5	5,3	13,0	4,9
	low	0%	1,7	4,6	1,4	65,4	0,1	0,0	0,0	18,4	4,2	7,2	3,7
	low	25%	33,5	17,4	2,5	104,2	0,2	57,0	1513,3	23,1	4,8	11,4	4,2
	low	50%	39,9	18,3	2,7	108,7	0,2	72,0	5575,8	23,3	4,8	12,6	4,2
	low	75%	43,6	19,4	2,9	110,8	0,3	82,0	7905,7	23,8	4,8	12,8	4,2
	low	100%	88,0	23,3	5,3	120,8	0,7	100,0	14187,3	24,5	5,3	15,0	4,9
	middle	0%	1,1	4,3	1,3	59,6	0,1	0,0	0,0	15,0	3,6	3,0	3,4
	middle	25%	15,5	16,1	1,8	92,7	0,2	40,0	600,0	21,3	4,6	9,2	4,0
**GREECE**	middle	50%	20,2	17,6	2,0	99,1	0,3	63,0	1860,1	22,1	4,8	11,0	4,3
	middle	75%	30,4	19,6	2,3	106,3	0,4	78,0	3238,8	22,8	5,0	13,2	4,8
	middle	100%	79,1	24,9	4,6	124,2	0,9	100,0	15929,8	24,5	5,4	17,0	5,4
	high	0%	0,8	2,5	1,3	71,3	0,1	0,0	0,0	12,9	3,5	1,7	3,4
	high	25%	9,8	14,7	1,6	93,2	0,3	2,0	989,9	20,1	4,6	8,1	4,0
	high	50%	13,9	16,3	1,7	98,4	0,4	42,0	3275,7	21,4	4,8	9,8	4,5
	high	75%	17,3	18,3	1,9	106,2	0,5	66,0	6184,7	22,3	5,1	12,4	4,9
	high	100%	51,1	24,7	3,5	128,6	0,9	100,0	23265,9	24,5	5,5	17,0	6,9
	low	0%	9,7	13,3	1,3	70,7	0,2	0,0	0,0	15,0	4,4	2,2	6,1
	low	25%	19,8	16,1	1,5	83,9	0,5	34,0	761,6	15,3	4,4	2,6	6,1
	low	50%	22,9	16,6	1,6	85,5	0,5	49,0	1337,9	15,4	4,5	2,6	6,2
	low	75%	25,8	17,0	1,9	86,4	0,6	61,0	2920,6	15,5	4,5	2,7	6,2
	low	100%	45,4	19,0	2,9	89,2	0,8	100,0	10647,1	15,7	4,5	2,9	6,2
	middle	0%	10,5	11,8	1,3	69,2	0,2	0,0	0,0	14,8	4,3	2,1	6,1
	middle	25%	19,9	16,1	1,5	83,7	0,4	35,0	700,0	15,3	4,4	2,6	6,1
**KORA**	middle	50%	22,9	16,7	1,6	85,4	0,5	50,0	1208,3	15,3	4,5	2,6	6,1
	middle	75%	26,4	17,0	1,9	86,5	0,6	62,0	2193,2	15,4	4,5	2,7	6,2
	middle	100%	47,4	19,2	3,1	89,2	0,8	100,0	10917,9	15,8	4,5	2,9	6,2
	high	0%	11,2	13,3	1,3	65,4	0,2	0,0	0,0	15,0	4,4	2,2	6,1
	high	25%	20,3	16,3	1,5	82,9	0,4	34,0	670,8	15,3	4,4	2,6	6,1
	high	50%	23,6	16,7	1,7	85,2	0,5	50,0	1168,3	15,3	4,5	2,6	6,1
	high	75%	27,7	17,1	2,0	86,5	0,6	61,0	1928,1	15,4	4,5	2,6	6,2
	high	100%	53,7	19,3	3,4	89,4	0,8	100,0	10062,3	15,7	4,5	2,9	6,2
	low	0%	7,8	8,9	0,6	25,5	0,1	0,0	0,0	14,6	3,2	3,3	4,4
	low	25%	28,2	16,4	1,4	72,2	0,4	44,0	509,9	15,1	3,8	4,2	5,4
**EPIC-NL**	low	50%	32,6	17,5	1,6	75,6	0,5	62,0	948,7	15,4	4,1	4,5	5,6
	low	75%	37,2	18,4	1,8	78,4	0,6	76,0	1552,4	15,7	4,4	4,7	5,8
	low	100%	86,8	24,5	4,1	86,2	0,8	100,0	9976,5	16,2	4,6	5,6	6,2
	middle	0%	8,3	8,9	0,6	25,5	0,1	0,0	0,0	14,6	3,2	3,3	4,4

	middle	25%	26,7	16,2	1,3	73,4	0,5	38,0	538,5	15,1	3,8	4,1	5,4
	middle	50%	31,0	17,3	1,5	76,3	0,5	57,0	1019,8	15,4	4,1	4,4	5,7
	middle	75%	35,1	18,2	1,7	78,8	0,6	72,0	1700,0	15,7	4,4	4,7	5,8
	middle	100%	86,8	24,5	4,1	86,3	0,8	100,0	10113,9	16,2	4,6	5,6	6,2
	high	0%	8,3	9,1	0,6	25,5	0,1	0,0	0,0	14,6	3,2	3,3	4,4
	high	25%	26,8	16,2	1,4	73,2	0,5	35,0	538,5	15,1	3,8	4,2	5,4
	high	50%	31,2	17,2	1,5	76,0	0,5	55,0	1019,8	15,4	4,1	4,5	5,6
	high	75%	35,4	18,1	1,7	78,6	0,6	71,0	1697,1	15,7	4,4	4,8	5,8
	high	100%	86,8	24,2	4,1	86,2	0,8	100,0	10046,4	16,2	4,6	5,6	6,2
	low	0%	11,6	12,2	0,9	44,6	0,3	0,0	0,0	14,7	3,7	3,5	5,2
	low	25%	25,0	16,1	1,3	50,5	0,5	32,0	565,7	15,2	3,9	4,1	5,4
	low	50%	29,9	17,2	1,5	53,8	0,5	47,0	1019,8	15,4	4,0	4,3	5,7
	low	75%	33,5	17,8	1,7	57,8	0,6	60,0	1749,3	15,4	4,2	4,7	5,7
	low	100%	50,8	19,9	2,6	65,7	0,8	91,0	5201,0	15,7	4,4	5,1	6,1
	middle	0%	11,6	12,8	0,9	44,9	0,2	0,0	0,0	14,7	3,6	3,4	5,1
	middle	25%	23,2	15,3	1,3	52,2	0,5	25,0	600,0	15,0	3,9	3,8	5,5
**PIAMA**	middle	50%	28,4	17,0	1,5	54,5	0,5	45,0	1077,0	15,4	4,1	4,2	5,7
	middle	75%	32,6	17,7	1,7	61,1	0,6	58,0	1841,2	15,4	4,2	4,5	5,8
	middle	100%	76,7	20,2	3,3	65,8	0,8	89,0	6140,0	16,0	4,5	5,2	6,2
	high	0%	11,9	12,5	0,9	44,3	0,2	0,0	0,0	14,7	3,6	3,4	5,0
	high	25%	24,6	15,6	1,3	51,7	0,5	27,0	565,7	15,0	4,0	4,0	5,5
	high	50%	29,3	17,2	1,5	54,0	0,5	45,0	1019,8	15,4	4,1	4,2	5,7
	high	75%	33,4	17,7	1,7	59,9	0,6	60,0	1868,2	15,4	4,2	4,3	5,8
	high	100%	52,9	19,5	2,6	66,2	0,8	91,0	6177,4	15,8	4,4	5,3	6,1
	low	0%	9,6	11,6	1,4	69,7	0,1	0,0	0,0	18,9	4,3	8,2	4,0
	low	25%	27,8	16,1	1,9	93,6	0,3	52,0	1019,8	20,3	4,5	9,8	4,2
	low	50%	31,9	16,6	2,2	95,4	0,4	69,0	2102,4	20,3	4,7	10,2	4,4
	low	75%	35,5	17,2	2,3	97,2	0,4	82,0	3712,1	20,4	4,8	10,4	4,5
	low	100%	65,8	20,8	3,9	104,8	0,9	100,0	11335,8	20,8	5,0	11,7	4,6
	middle	0%	10,4	11,6	1,4	57,3	0,1	0,0	0,0	18,9	4,3	8,2	4,0
	middle	25%	28,1	16,1	2,0	93,3	0,3	50,0	1000,0	20,3	4,5	9,8	4,2
**Rome**	middle	50%	32,5	16,6	2,2	95,2	0,4	68,0	1910,5	20,3	4,7	10,2	4,4
	middle	75%	36,1	17,2	2,4	97,0	0,4	82,0	3512,8	20,4	4,8	10,4	4,5
	middle	100%	87,5	21,4	4,9	104,8	0,8	100,0	11428,0	20,8	5,0	11,7	4,6
	high	0%	10,5	11,6	1,4	70,6	0,1	0,0	0,0	18,9	4,3	8,2	4,0
	high	25%	30,2	16,2	2,1	92,7	0,3	51,0	854,4	20,3	4,5	10,2	4,3
	high	50%	34,0	16,8	2,3	94,5	0,3	70,0	1615,5	20,4	4,7	10,2	4,4
	high	75%	37,7	17,4	2,4	96,3	0,4	83,0	3000,0	20,4	4,7	10,4	4,4
	high	100%	65,8	20,8	3,9	104,8	0,8	100,0	11442,5	20,8	5,0	11,7	4,6
	low	0%	1,8	0,4	0,8	59,6	0,2	0,0	0,0	2,3	3,9	-9,8	5,8
	low	25%	17,3	13,9	1,4	91,4	0,5	23,0	608,3	13,5	4,5	2,1	6,3
	low	50%	21,8	15,8	1,6	94,8	0,5	43,0	1456,0	15,2	4,5	3,8	6,5
	low	75%	26,9	17,1	1,8	99,7	0,6	60,0	3061,0	16,1	4,6	4,6	6,7
	low	100%	73,4	28,0	4,4	116,2	0,9	100,0	14641,4	18,9	5,2	6,9	8,0
	middle	0%	1,8	2,0	0,9	56,2	0,2	0,0	0,0	2,7	3,9	-9,6	5,8
	middle	25%	18,4	14,7	1,4	91,1	0,5	24,0	583,1	14,4	4,5	3,0	6,3
**SNC**	middle	50%	22,2	16,1	1,6	94,2	0,6	42,0	1345,4	15,7	4,5	4,3	6,5
	middle	75%	26,9	17,3	1,8	98,5	0,6	57,0	2657,1	16,2	4,6	4,7	6,6
	middle	100%	82,9	28,0	4,9	115,9	0,9	100,0	12983,8	18,9	5,1	6,9	8,0
	high	0%	2,0	3,1	0,9	56,2	0,0	0,0	0,0	2,7	3,9	-9,6	5,8
	high	25%	20,7	15,6	1,5	90,5	0,5	26,0	447,2	15,3	4,5	3,9	6,2
	high	50%	24,5	16,6	1,7	93,1	0,6	42,0	1000,0	15,8	4,5	4,5	6,3
	high	75%	29,3	17,6	1,9	96,9	0,6	56,0	2147,1	16,2	4,5	4,7	6,5
	high	100%	80,3	25,5	4,6	116,1	0,9	100,0	11704,7	18,9	5,1	6,9	7,9
	low	0%	0,1	-0,9	-0,5	46,3	-0,1	0,0	0,0	3,0	4,4	-13,1	4,4
	low	25%	9,7	6,4	0,3	74,6	0,5	0,0	400,0	12,4	5,0	-3,9	5,9
	low	50%	14,4	8,0	0,6	77,4	0,5	13,0	806,2	12,9	5,3	-2,8	6,3
	low	75%	19,5	9,4	0,8	80,4	0,6	34,0	1486,6	13,2	5,5	-1,6	6,6
**SWEDEN**	low	100%	69,7	23,4	3,1	91,4	0,9	100,0	9552,5	13,8	6,1	1,4	8,6
	middle	0%	0,1	-1,2	-0,5	49,3	-0,1	0,0	0,0	3,0	4,4	-13,0	4,4
	middle	25%	11,3	6,9	0,4	74,5	0,5	1,0	360,6	12,6	5,1	-3,7	5,9
	middle	50%	16,2	8,3	0,7	77,2	0,5	15,0	761,6	12,9	5,4	-2,7	6,2
	middle	75%	21,9	9,7	0,9	80,0	0,6	37,0	1403,6	13,2	5,6	-1,6	6,5

	middle	100%	69,7	23,4	3,1	91,4	0,9	100,0	9552,5	13,8	6,1	1,4	8,6
	high	0%	0,2	0,3	-0,5	50,5	-0,1	0,0	0,0	3,0	4,4	-12,1	4,4
	high	25%	13,7	7,6	0,5	74,2	0,4	4,0	400,0	12,9	5,1	-3,7	6,0
	high	50%	19,1	8,6	0,8	76,7	0,5	20,0	806,2	13,0	5,5	-2,7	6,2
	high	75%	24,5	10,9	1,0	79,7	0,6	44,0	1600,0	13,2	5,6	-1,5	6,4
	high	100%	64,7	23,4	3,0	91,4	0,9	100,0	9123,6	13,8	6,1	1,4	8,6

Supplementary Table S4: Variance explained by the first PCs, by cohort and domain.

	**Variance explained (%)**					
	**Ambient air pollution**		**PC1 – Built Environment**		**PC1 – Air Temperature**	
**Cohort**	PC1	PC2	PC1	PC2	PC1	PC2
**Switzerland (SNC)**	79.1	9.9	61.7	28.4	74.1	20.6
**ROME**	83.3	9.4	57.5	32.4	76.1	23.3
**GREECE**	62.0	27.3	60.4	33.4	67.9	24.5
**SWEDEN (SIMSAM)**	64.9	28.2	58.8	32.4	67.3	23.1
**CATALONIA**	92.1	4.7	58.1	31.6	69.3	28.7
**EPIC-NL**	72.6	17.0	57.4	29.8	78.5	20.6
**AMIGO**	78.2	14.9	55.4	30.6	72.9	27.1
**GCAT**	78.3	11.5	64.1	33.2	25.4	25.1
**KORA**	82.0	13.7	64.4	24.0	70.4	27.3
**CEANS**	74.4	14.6	59.4	31.8	91.0	7.7
**BAMSE**	73.0	12.6	74.7	24.1	83.4	15.2
**PIAMA**	82.4	10.3	66.9	28.6	59.5	39.3

Supplementary figures

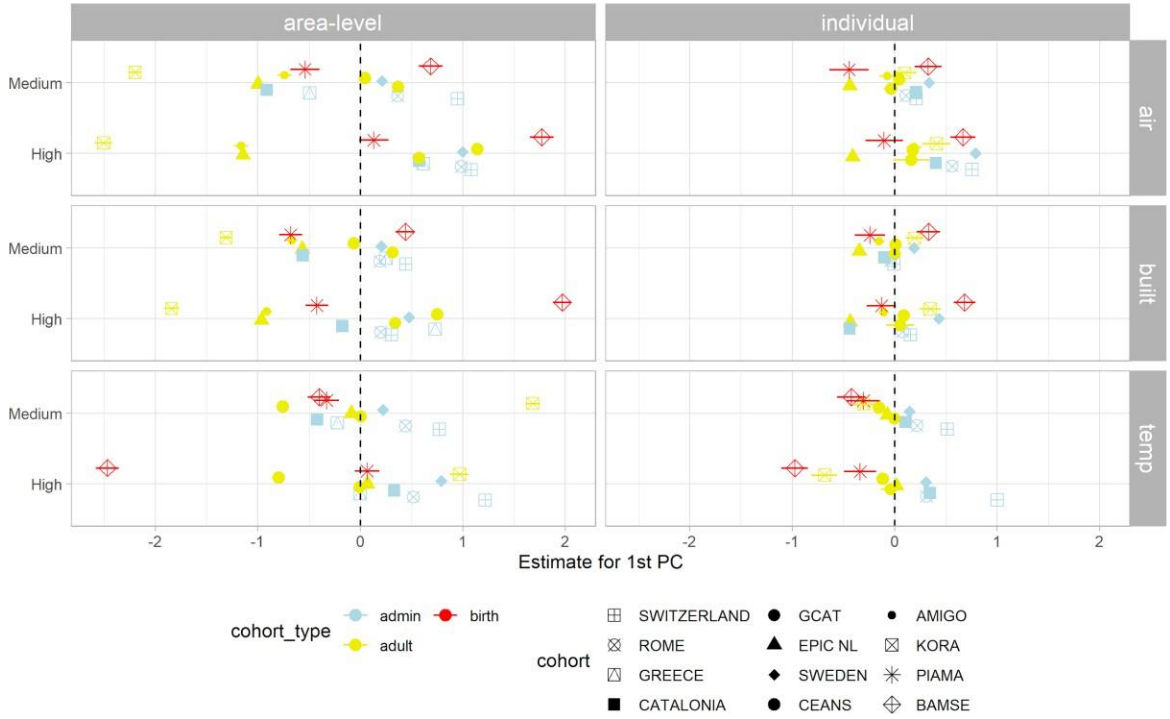

Supplementary Figure S1: Association between the first PC of each domain of the external exposome and individual- and area-level SEP. Coefficient estimates are reported with 95% confidence intervals from the multivariable linear models adjusting for age (except birth cohorts). Estimates on the right side of the vertical dotted line represent a positive association between SEP and the first PC of each external exposome domain (e.g. higher levels of traffic-related pollution, built up land use, and warm season mean temperature associated with higher SEP). Results are displayed with separate colors according to cohort type.

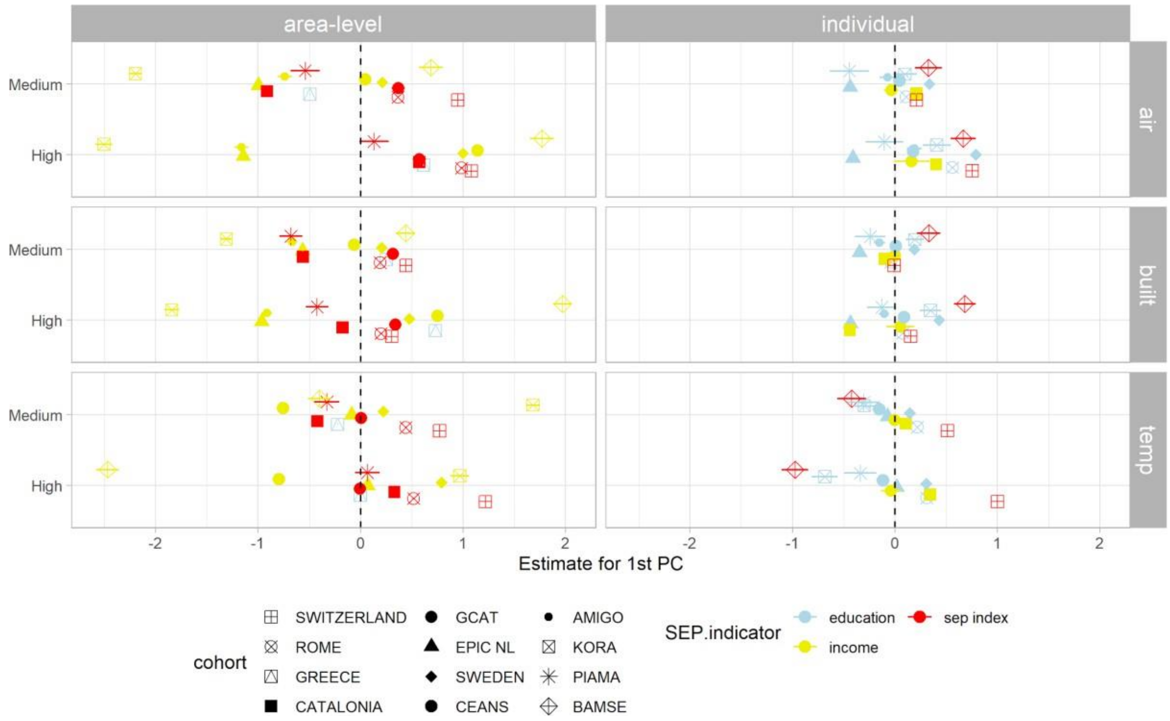

Supplementary Figure S2: Association between the first PC of each domain of the external exposome and individual- and area-level SEP. Coefficient estimates are reported with 95% confidence intervals from the multivariable linear models adjusting for age (except birth cohorts). Estimates on the right side of the vertical dotted line represent a positive association between SEP and the first PC of each external exposome domain (e.g. higher levels of traffic-related pollution, built up land use, and warm season mean temperature associated with higher SEP). Results are displayed with separate colors according to SEP indicator type.

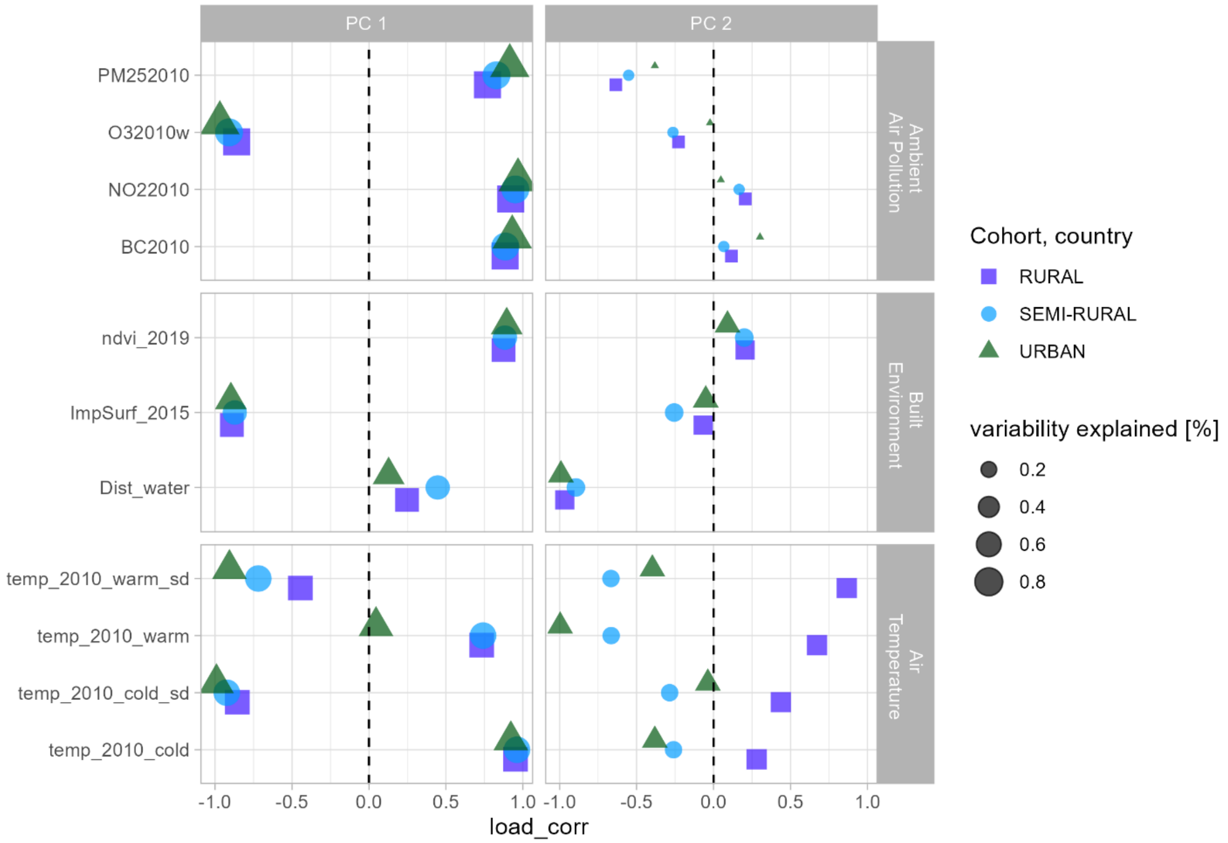

Supplementary Figure S3: Distribution of PCA loadings by cohort and external exposome domain in the Catalan administrative cohort, stratified by urbanicity groups. Symbol size is proportional to PC contribution of the exposure variables.

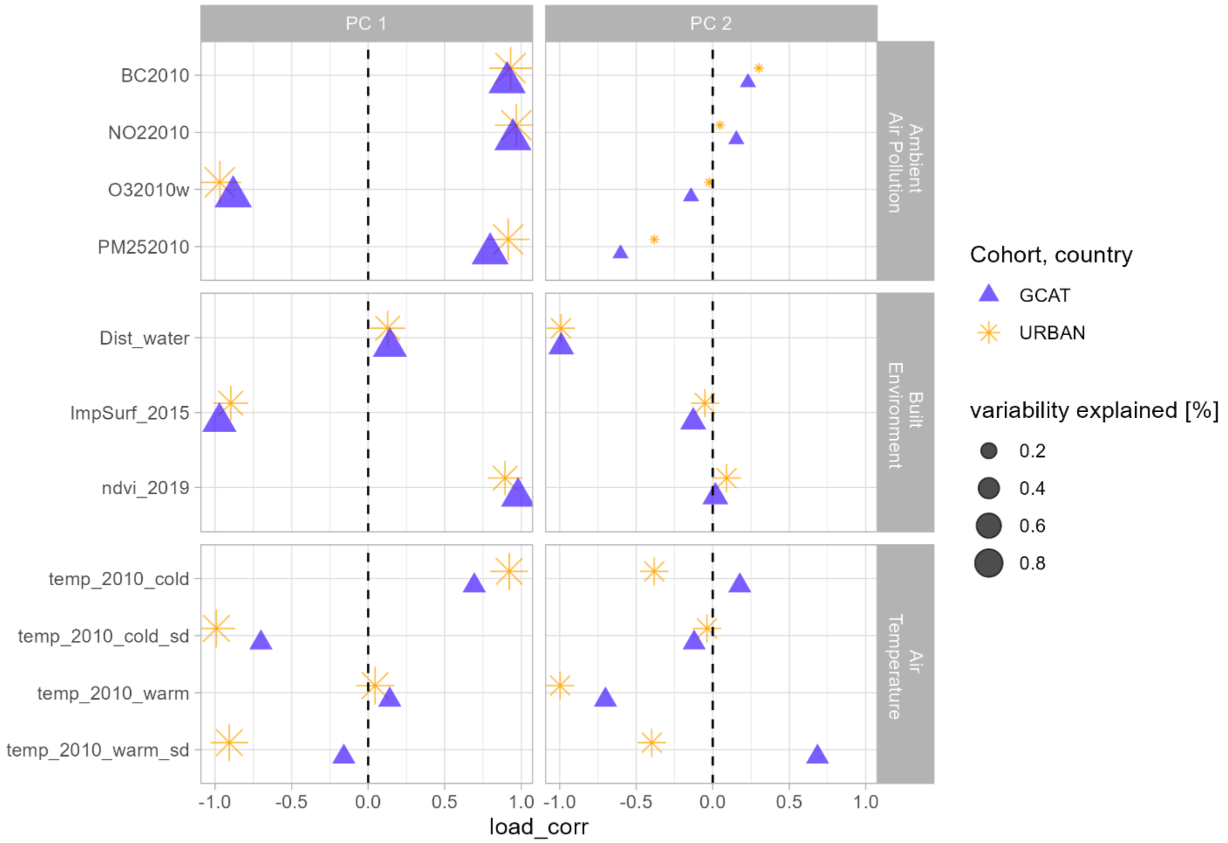

Supplementary Figure S4: Distribution of PCA loadings by cohort and external exposome domain in GCAT and the urban portion of the Catalan administrative cohort. Symbol size is proportional to PC contribution of the exposure variables.

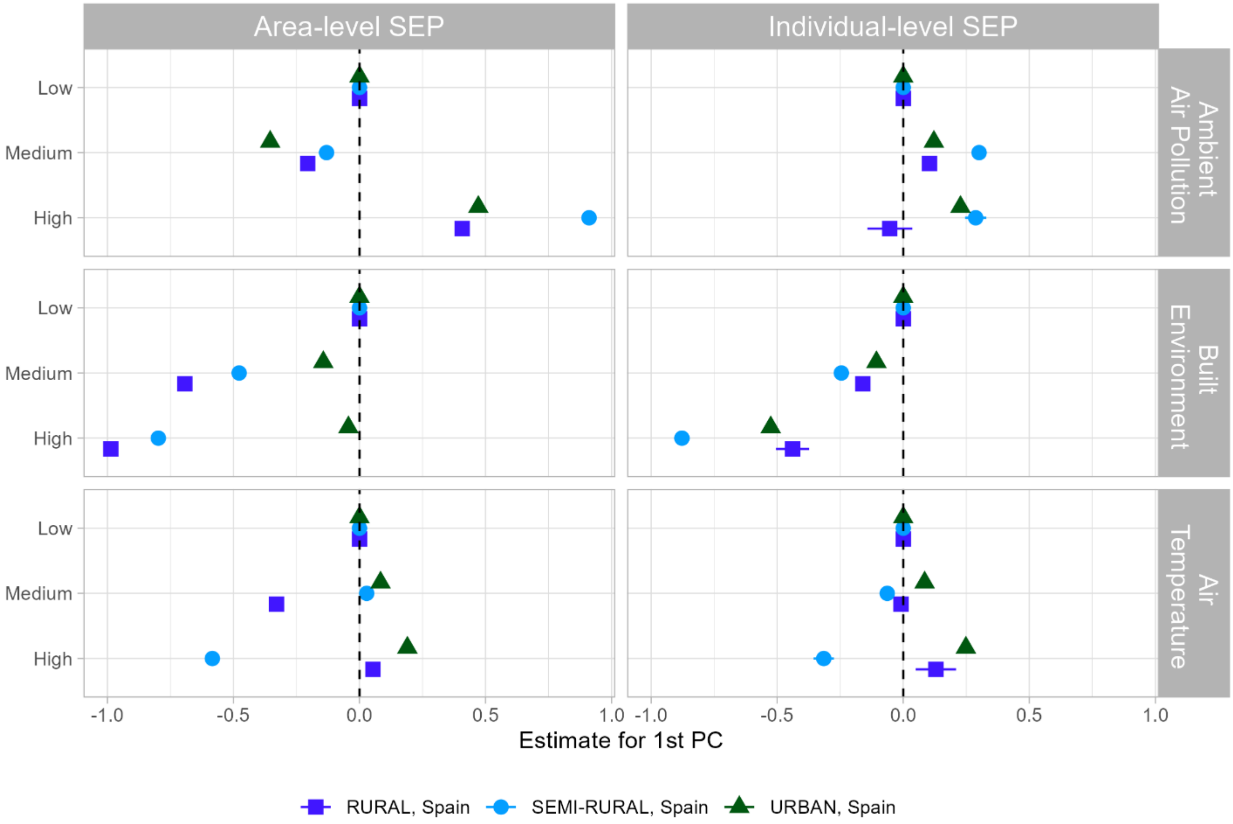

Supplementary Figure S5: Association between the **first PC** of each domain of the external exposome and individual- and area-level SEP **in the Catalan administrative cohort**, **stratified by urbanicity group.** Coefficient estimates are reported with 95% confidence intervals from the multivariable linear models adjusting for age (except birth cohorts). Estimates on the right side of the vertical dotted line represent a positive association between SEP and the first PC of each external exposome domain.

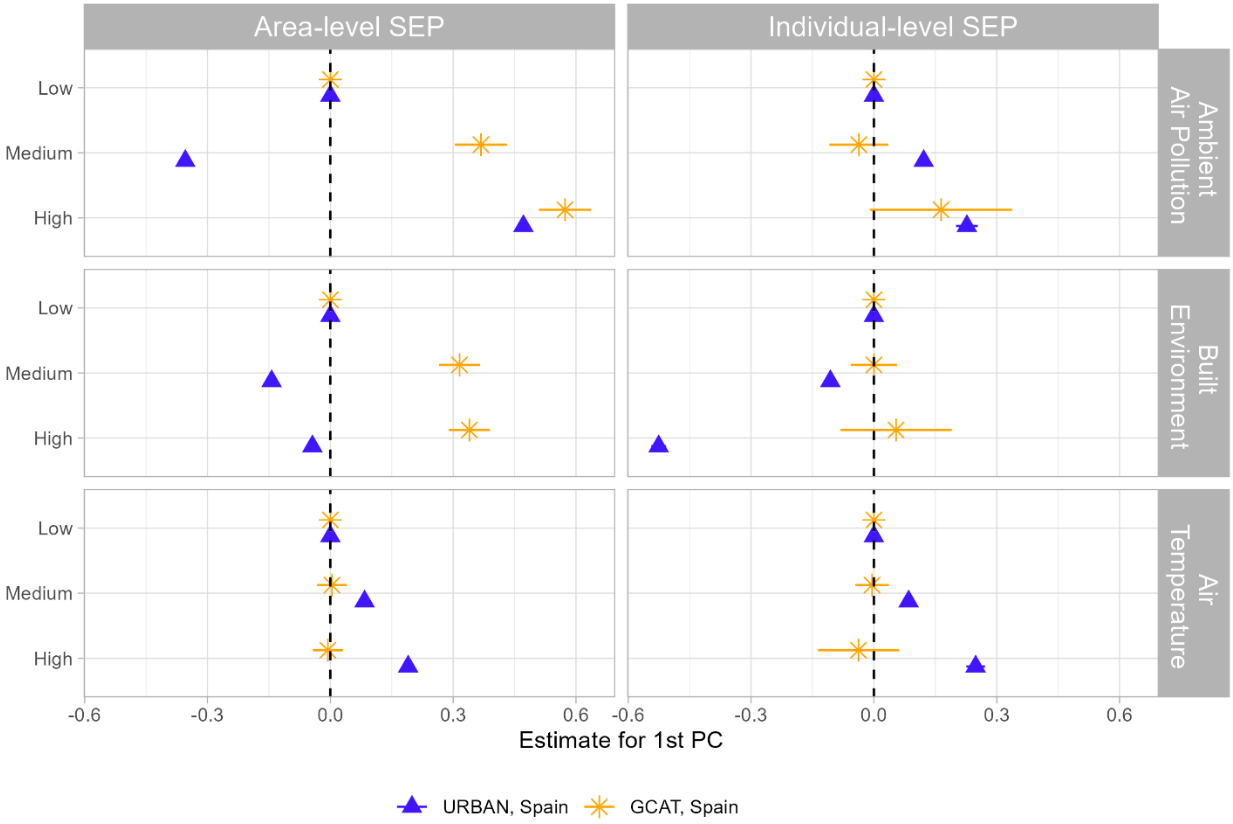

Supplementary Figure S6: Association between the **first PC** of each domain of the external exposome and individual- and area-level SEP **in the GCAT cohort and the urban subset of the Catalan administrative cohort.** Coefficient estimates are reported with 95% confidence intervals from the multivariable linear models adjusting for age (except birth cohorts). Estimates on the right side of the vertical dotted line represent a positive association between SEP and the first PC of each external exposome domain.

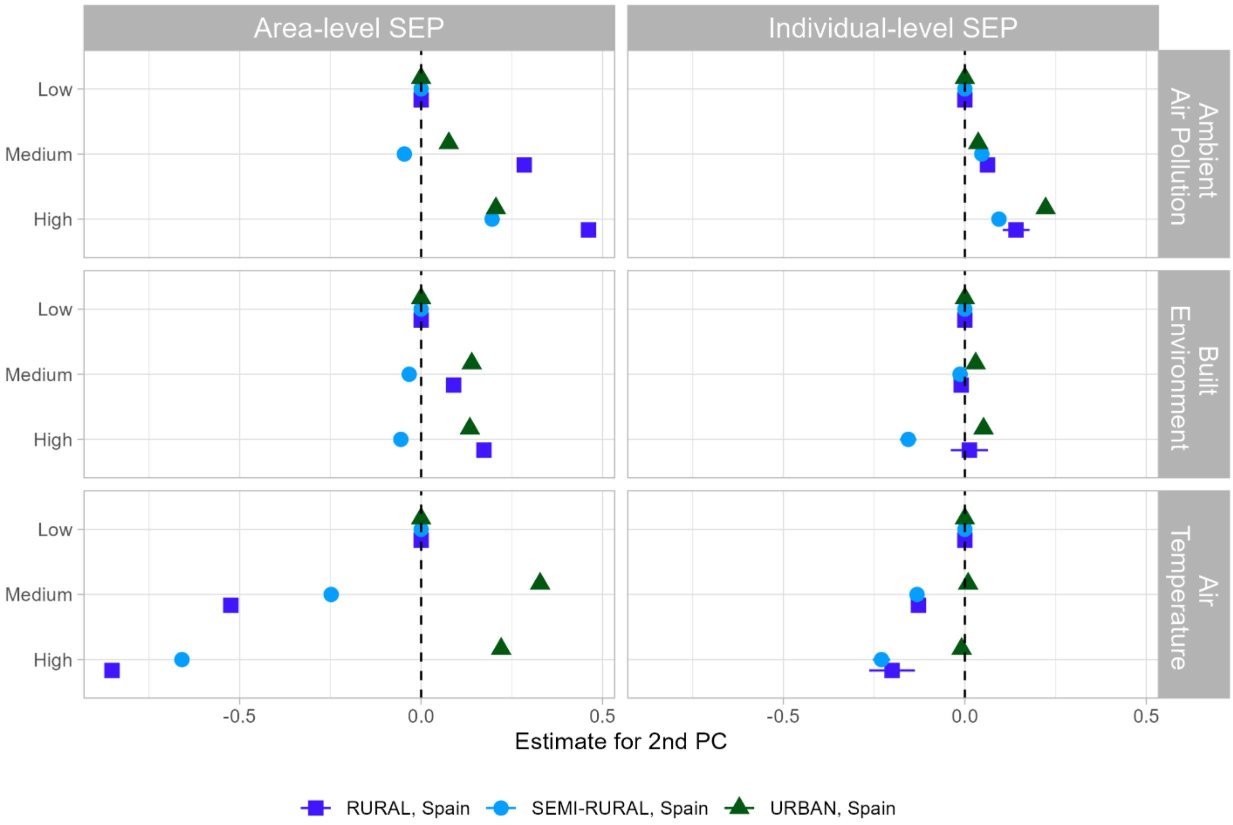

Supplementary Figure S7: Association between the **second PC** of each domain of the external exposome and individual- and area-level SEP in the **Catalan administrative cohort, stratified by urbanicity group.** Coefficient estimates are reported with 95% confidence intervals from the multivariable linear models adjusting for age (except birth cohorts). Estimates on the right side of the vertical dotted line represent a positive association between SEP and the first PC of each external exposome domain.

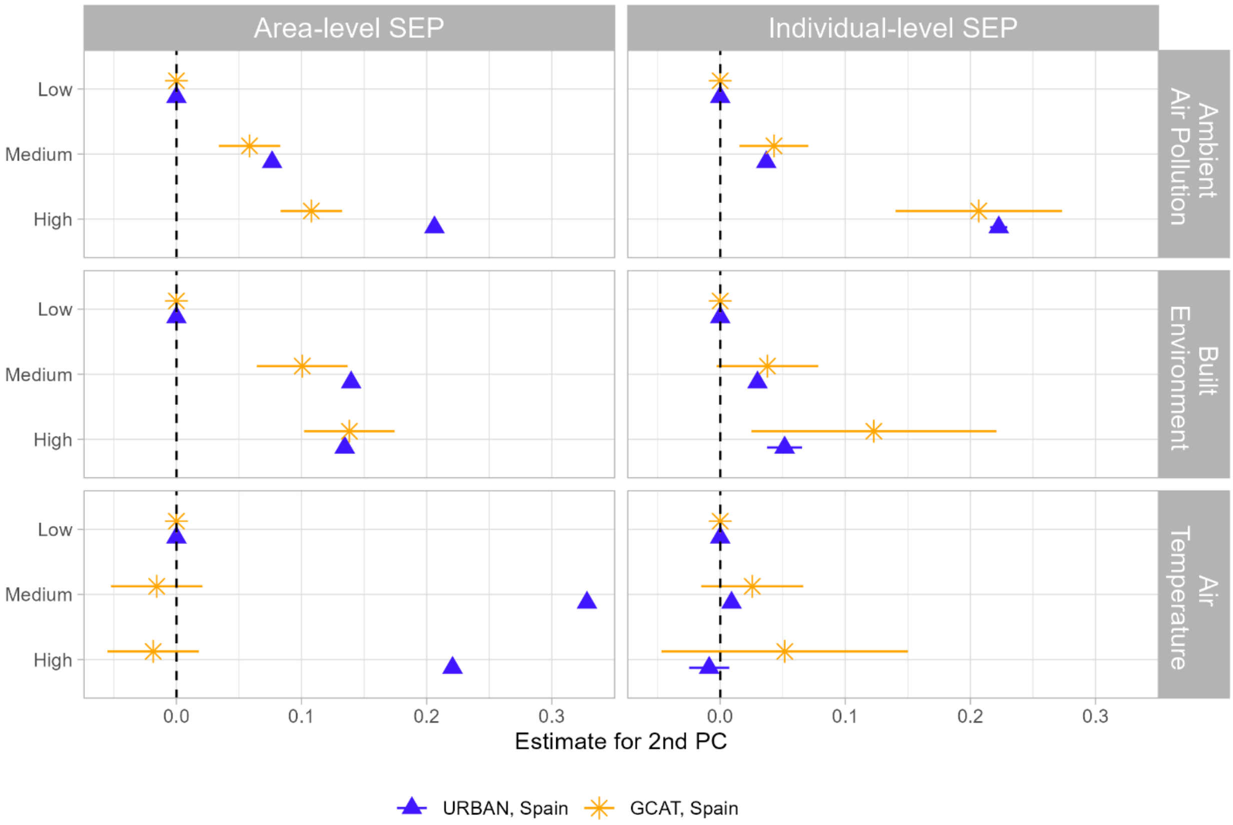

Supplementary Figure S8: Association between the **second PC** of each domain of the external exposome and individual- and area-level SEP **in the GCAT cohort and the urban subset of the Catalan administrative cohort.** Coefficient estimates are reported with 95% confidence intervals from the multivariable linear models adjusting for age (except birth cohorts). Estimates on the right side of the vertical dotted line represent a positive association between SEP and the first PC of each external exposome domain.

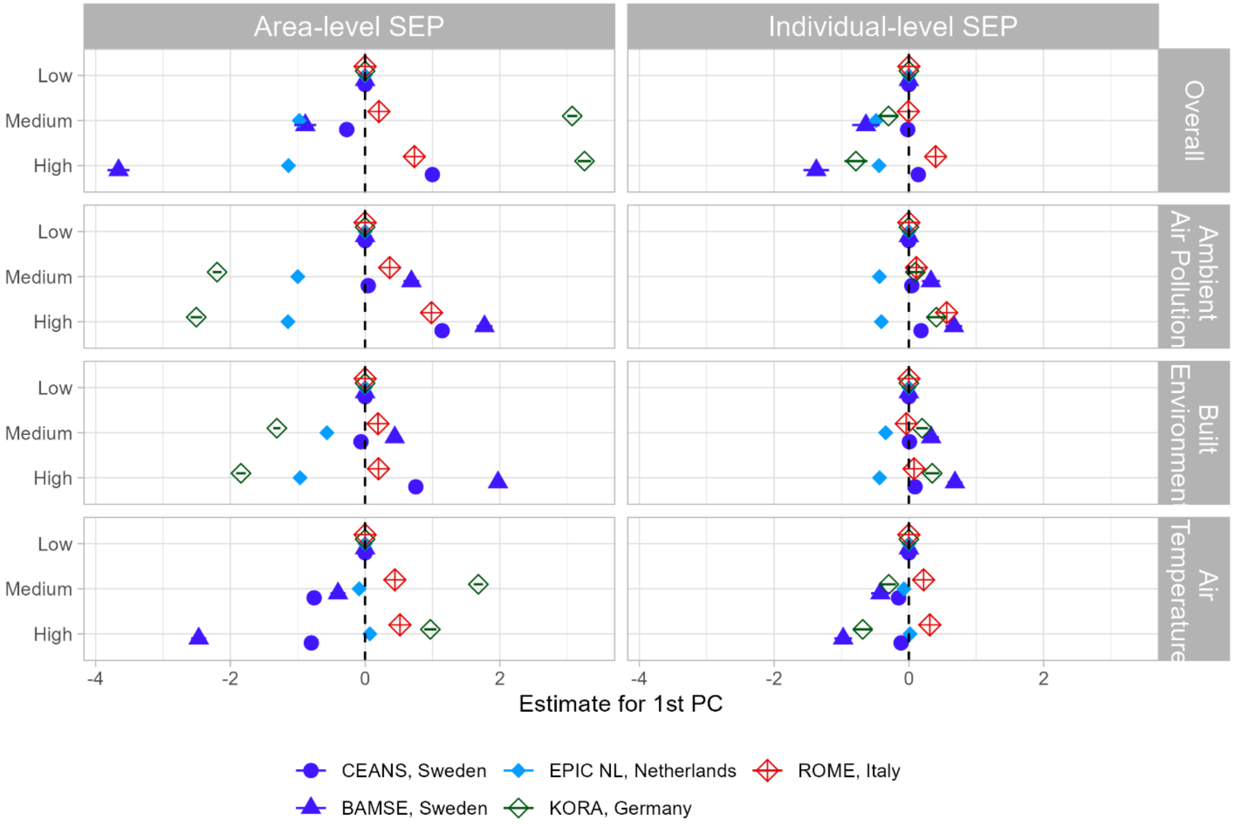

Supplementary Figure S9: Association between the first PC of each domain of the external exposome and individual and area-level SEP, restricted to cohorts with **city or municipality** coverage. Coefficient estimates are reported with 95% confidence intervals from the multivariable linear models adjusting for age (except birth cohorts). Estimates on the right side of the vertical dotted line represent a positive association between SEP and the first PC of each external exposome domain (e.g. higher levels of traffic-related pollution, built up land use, and warm season mean temperature associated with higher SEP).

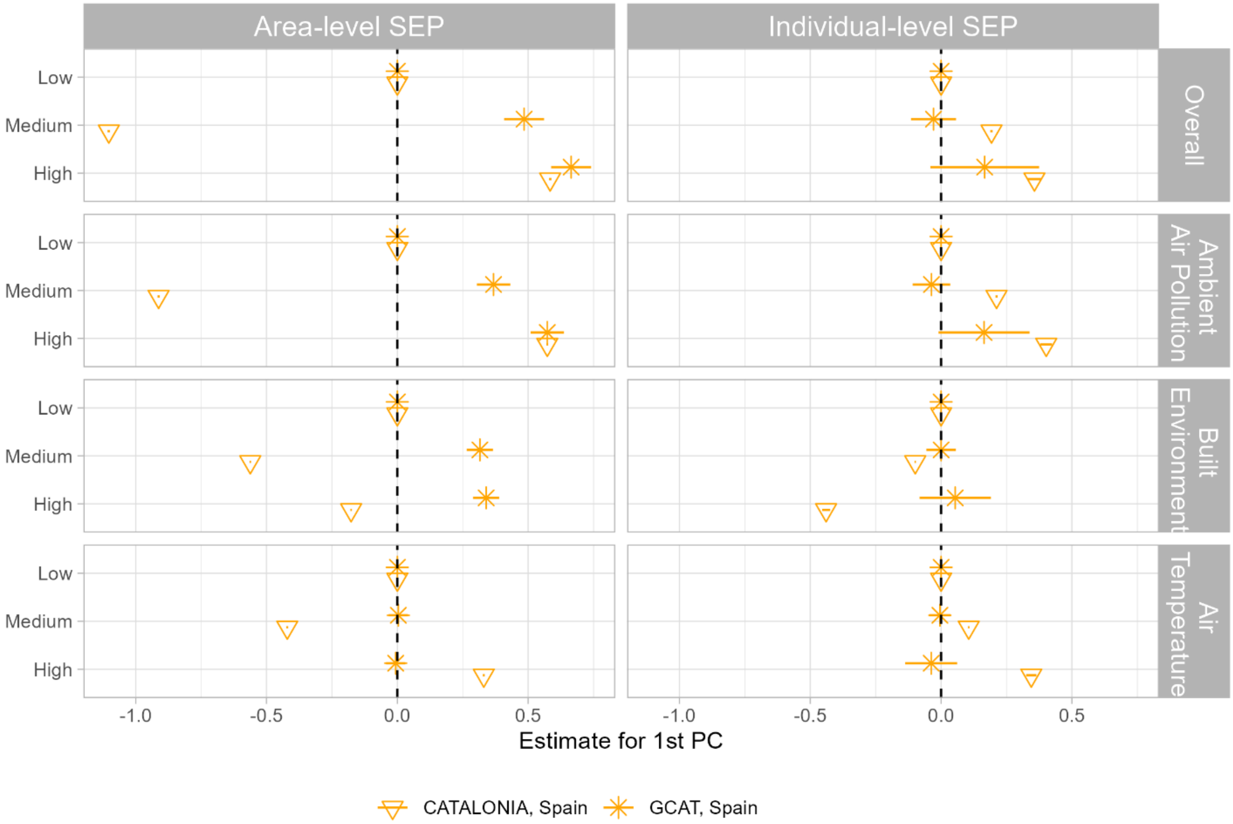

Supplementary Figure S10: Association between the first PC of each domain of the external exposome and individual and area-level SEP, restricted to cohorts with **regional** coverage. Coefficient estimates are reported with 95% confidence intervals from the multivariable linear models adjusting for age (except birth cohorts). Estimates on the right side of the vertical dotted line represent a positive association between SEP and the first PC of each external exposome domain (e.g. higher levels of traffic-related pollution, built up land use, and warm season mean temperature associated with higher SEP).

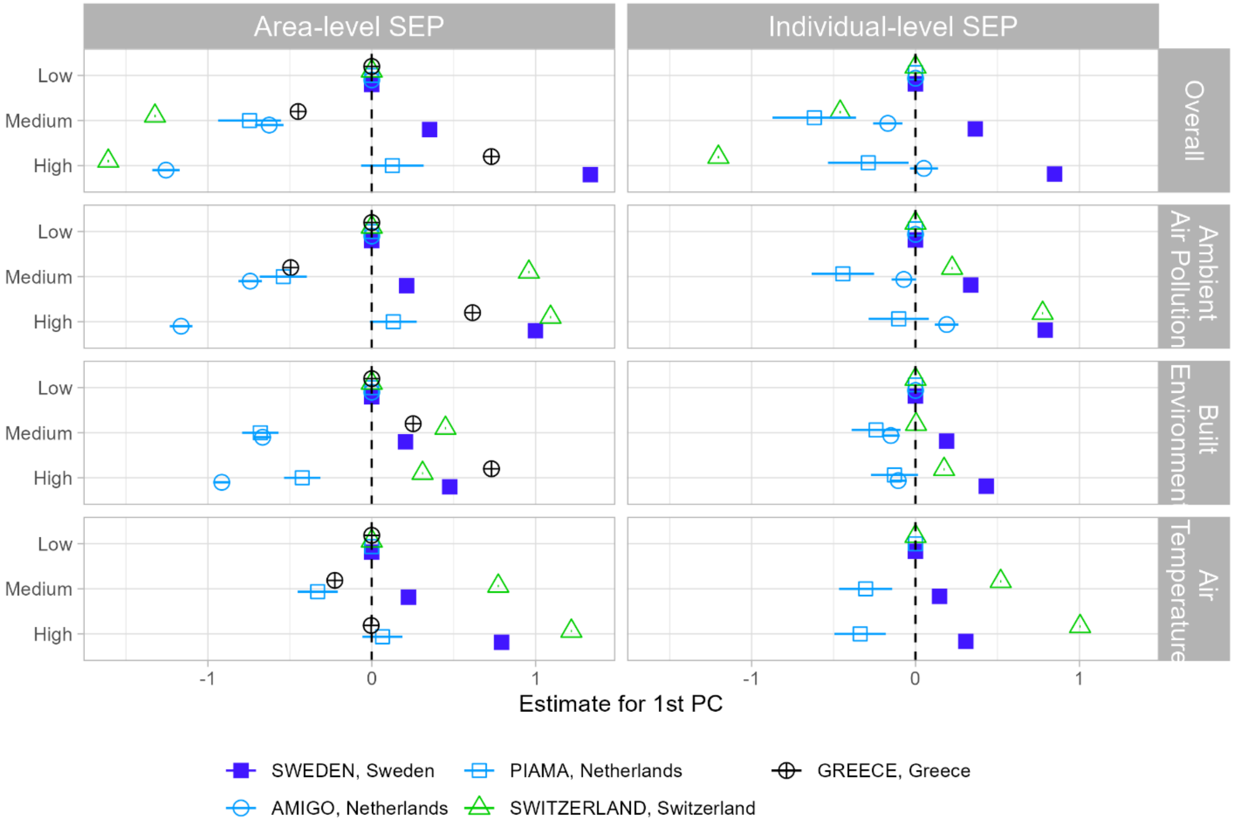

Supplementary Figure S11: Association between the first PC of each domain of the external exposome and individual and area-level SEP, restricted to cohorts with **country-wide** coverage. Coefficient estimates are reported with 95% confidence intervals from the multivariable linear models adjusting for age (except birth cohorts). Estimates on the right side of the vertical dotted line represent a positive association between SEP and the first PC of each external exposome domain (e.g. higher levels of traffic-related pollution, built up land use, and warm season mean temperature associated with higher SEP).

References

1. Lichtenstein, P. *et al.* The Swedish Twin Registry in the Third Millennium: An Update. *Twin Res. Hum. Genet.* **9**, 875–882 (2006).
2. Eriksson, A. K. *et al.* Psychological distress and risk of pre-diabetes and Type 2 diabetes in a prospective study of Swedish middle-aged men and women. *Diabet. Med.* **25**, 834–842 (2008).
3. Wändell, P. E., Wajngot, A., de Faire, U. & Hellénius, M. L. Increased prevalence of diabetes among immigrants from non-European countries in 60-year-old men and women in Sweden. *Diabetes Metab.* **33**, 30–36 (2007).
4. Lagergren, M. *et al.* A longitudinal study integrating population, care and social services data. The Swedish National study on Aging and Care (SNAC). *Aging Clin. Exp. Res.* **16**, 158–168 (2004).
5. Spoerri, A., Zwahlen, M., Egger, M. & Bopp, M. The Swiss National Cohort: A unique database for national and international researchers. *Int. J. Public Health* **55**, 239–242 (2010).
6. Bopp, M. *et al.* Cohort Profile: The Swiss National Cohort—a longitudinal study of 6.8 million people. *Int. J. Epidemiol.* **38**, 379–384 (2009).
7. Panczak, R. *et al.* A Swiss neighbourhood index of socioeconomic position: development and association with mortality. *J. Epidemiol. Community Health* **66**, 1129–1136 (2012).
8. Panczak, R., Berlin, C., Voorpostel, M., Zwahlen, M. & Egger, M. The Swiss neighbourhood index of socioeconomic position: update and re-validation. *Swiss Med. Wkly.* **153**, 40028–40028 (2023).
9. Beulens, J. W. J. *et al.* Cohort Profile: The EPIC-NL study. *Int. J. Epidemiol.* **39**, 1170–1178 (2010).
10. Central Bureau of Statistics (CBS). District and neighbourhood map 2015. *The Netherlands* (2022).
11. Slottje, P., Yzermans, C. J., Korevaar, J. C., Hooiveld, M. & Vermeulen, R. C. H. The population-based Occupational and Environmental Health Prospective Cohort Study (AMIGO) in the Netherlands. *BMJ Open* **4**, e005858 (2014).
12. Cesaroni, G. *et al.* Long-term exposure to urban air pollution and mortality in a cohort of more than a million adults in Rome. *Environ. Health Perspect.* **121**, 324–331 (2013).
13. Cesaroni, G. *et al.* Mortality inequalities in Rome: The role of individual education and neighbourhood real estate market. *Epidemiol. Prev.* **44**, 31–37 (2020).
14. Cesaroni, G. *et al.* Socioeconomic position and health status of people who live near busy roads: The Rome Longitudinal Study (RoLS). *Environ. Heal. A Glob. Access Sci. Source* **9**, 1–12 (2010).
15. Wijga, A. H. *et al.* Cohort profile: The Prevention and Incidence of Asthma and Mite Allergy (PIAMA) birth cohort. *Int. J. Epidemiol.* **43**, 527–535 (2014).
16. Bajekal, M., Jan, S. & Jarman, B. The Swedish UPA score. *http://dx.doi.org/10.1177/140349489602400309* **24**, 177–183 (2016).
17. Helmoltz Munich. KORA cohort (Cooperative Health Research in the Region of Augsburg) -

Helmholtz Munich. https://www.helmholtz-munich.de/en/epi/cohort/kora (2023).

18. Holle, R., Happich, M., Löwel, H. & Wichmann, H. E. KORA - A research platform for population based health research. *Gesundheitswesen* **67**, (2005).

19. Ranzani, O. *et al.* Long-term exposure to air pollution and severe COVID-19 in Catalonia: a population-based cohort study. *Nat. Commun. 2023 141* **14**, 1–9 (2023).

20. Institut d'Estadística de Catalunya. Índex socioeconòmic territorial. Catalunya. https://www.idescat.cat/pub/?id=ist (2023).

21. Obón-Santacana, Mireia, et al. "GCAT| Genomes for life: a prospective cohort study of the genomes of Catalonia*." BMJ open 8.3* (2018): e018324.
